# Casirivimab + imdevimab accelerates symptom resolution linked to improved COVID-19 outcomes across susceptible antibody and risk profiles

**DOI:** 10.1038/s41598-023-39681-7

**Published:** 2023-08-07

**Authors:** Dateng Li, Meng Xu, Andrea T. Hooper, Diana Rofail, Kusha A. Mohammadi, Yiziying Chen, Shazia Ali, Thomas Norton, David M. Weinreich, Bret J. Musser, Jennifer D. Hamilton, Gregory P. Geba

**Affiliations:** Global Development, Regeneron Pharmaceuticals, Inc., 777 Old Saw Mill River Road, Tarrytown, NY 10591 USA

**Keywords:** Immunology, Infectious diseases

## Abstract

Severe, protracted symptoms are associated with poor outcomes in severe acute respiratory syndrome coronavirus 2 (SARS-CoV-2) infection. In a placebo-controlled study of casirivimab and imdevimab (CAS + IMD) in persons at high risk of severe coronavirus disease 2019 (COVID-19; *n* = 3816), evolution of individual symptoms was assessed for resolution patterns across risk factors, and baseline SARS-CoV-2-specific antibody responses against S1 and N domains. CAS + IMD versus placebo provided statistically significant resolution for 17/23 symptoms, with greater response linked to absence of endogenous anti–SARS-CoV-2 immunoglobulin (Ig)G, IgA, or specific neutralizing antibodies at baseline, or high baseline viral load. Resolution of five key symptoms (onset days 3–5)—dyspnea, cough, feeling feverish, fatigue, and loss of appetite—independently correlated with reduced hospitalization and death (hazard ratio range: 0.31–0.56; *P* < 0.001–0.043), and was more rapid in CAS + IMD-treated patients lacking robust early antibody responses. Those who seroconverted late still benefited from treatment. Thus, highly neutralizing COVID-19-specific antibodies provided by CAS + IMD treatment accelerated key symptom resolution associated with hospitalization and death in those at high risk for severe disease as well as in those lacking early, endogenous neutralizing antibody responses.

## Introduction

The coronavirus disease 2019 (COVID-19) pandemic, which began in late 2019 in Wuhan, China, has led to more than 600 million infections and 6 million deaths^1^, and is caused by severe acute respiratory syndrome coronavirus 2 (SARS-CoV-2). This virus is an enveloped, single-stranded, RNA virus of moderate infectivity that is transmitted via respiratory aerosols^2,3^. SARS-CoV-2 infection affects individuals across age and racial groups with variable clinical impact, including asymptomatic carriage, upper respiratory symptoms of varying intensity, pneumonia, and acute respiratory distress syndrome culminating in ventilatory failure and death^4–8^. Globally, the most important risk factors for severe disease appear to be old age, obesity, and metabolic derangements, such as type 2 diabetes, coronary artery disease, and hypertension, as well as their intersections^8–12^.

As clinical experience increases, opportunities for understanding the relationship between COVID-19 symptoms and clinical outcomes are growing^13–20^. Early in the pandemic, non-specific upper respiratory symptoms of COVID-19 were difficult to distinguish from those associated with other respiratory viruses. A symptom initially noted to be distinctive (albeit not experienced by all patients) was loss of taste/smell; this was later found by some to be strongly associated with infection^17,21^. Studies of the immunological response in COVID-19 infection have now partly revealed a relationship with clinical outcomes^22–26^, but data linking the immune response to the evolution or resolution of symptoms are still inconclusive.

Innate and adaptive immune responses, and the attendant cytokine profile, are critical for the effective clearance of SARS-CoV-2. However, these inflammatory responses may drive symptoms, and a prolonged response may be responsible for the tissue damage observed in severe COVID-19^22^. Markers of aging-related and obesity-associated inflammation may play a role in severe COVID-19 via several innate immunity-related pathways^27–30^. Severe inflammation via these innate immune routes may account for the high rates and severity of symptoms noted in patients who are older, male, or who have obesity, and may also provide opportunities for directed therapy^31^. In contrast, adaptive immunity manifested as neutralizing antibodies (NAbs) are important for viral clearance, reduction in disease severity, and survival following a COVID-19 diagnosis^32–34^. However, these associations have not been investigated in a large clinical trial examining COVID-19 symptoms and outcomes upon treatment with anti-SARS-CoV-2 monoclonal antibodies.

CAS + IMD (REGEN-COV®), comprising a combination of two monoclonal antibodies (casirivimab and imdevimab) targeting the receptor-binding domain of the SARS-CoV-2 spike protein, was developed for the treatment or prevention of COVID-19. In combination, these two antibodies exhibited strong in vitro neutralization of SARS-CoV-2 to prevent disease in non-human primates, and to prevent rapid mutational escape observed with individual antibodies in vitro^35–37^. The efficacy and safety of CAS + IMD were evaluated in a large double-blind, randomized, seamless, clinical trial of more than 6000 outpatients with quantitative reverse transcriptase PCR (RT-qPCR)-confirmed SARS-CoV-2 infection^38^. Results from the Phase III portion of the trial, which was conducted prior to the widespread circulation of the Omicron variant, demonstrated that CAS + IMD reduced the risk of COVID-19–related hospitalization and death from any cause by approximately 70%^38^. The secondary analysis conducted herein was performed to assess the effects of CAS + IMD treatment on symptoms and clinical outcome as a function of COVID-19 risk factors and antibody repertoire at randomization in outpatients at high risk of severe disease.

## Results

### Study population and baseline symptoms

Overall, this secondary analysis involved 3816 patients (placebo: *n* = 1258; CAS + IMD combined dose group: *n* = 2558) with at least 1 risk factor for severe COVID-19 (Supplemental Table 1) from Phases I, II, and III of this clinical study. Patient demographic and baseline characteristics were balanced across treatment groups (Supplemental Table 2a). The most common risk factors for severe COVID-19 were obesity, defined as body mass index (BMI) ≥ 30 kg/m^2^ (59.1%), age ≥ 50 years (50%), and cardiovascular disease, including hypertension (35.2%) (Supplemental Table 2a). The rates of hospitalization/death were 3.8% (48/1258) for patients in the placebo group and 0.8% (20/2558; Supplemental Table 2b) for patients in the CAS + IMD group, a risk reduction in this expanded, pooled population that was consistent with previously reported results from the Phase III portion of the trial^38,39^. The outcome of hospitalization and/or death in different subgroups is shown in Supplemental Table 3.

All patients were symptomatic at baseline, and the median number of days of symptoms at the time of randomization was 3. The five most common symptoms at baseline were cough (71.6%), fatigue (64.8%), headache (61.2%), body aches (53.5%), and loss of taste/smell (44.9%) (Supplemental Table 2c).

### Antibody profile at baseline and during treatment

Antibodies were identified by class and ability to recognize the SARS-CoV-2 spike protein^40^. At baseline, 67.8% of patients (2589/3816) were seronegative for anti–SARS-CoV-2 antibodies (immunoglobulin [Ig]A [anti-spike; anti-S], IgG [anti-S], and IgG [anti-nucleocapsid; anti-N]), and 29.5% of patients (1124/3816) were seropositive for at least one of the three antibodies tested (Supplemental Table 2d). Of those who were seropositive, 1007 (89.6%), 427 (38.0%), and 453 (40.3%) were positive for IgA (anti-S), IgG (anti-S), and IgG (anti-N), respectively. Anti-S IgG and anti-N IgG showed a high concordance of 92.6% (Hooper et al. 2023, manuscript in preparation). Of patients who were seronegative at baseline, a total of 2291 with testing results available (88.5%), 1929 (84.2%) seroconverted and were seropositive for IgG (anti-N) by day 29; 362 (15.8%) patients remained seronegative (Supplemental Table 4).

Samples from the 1124 seropositive patients were tested for NAbs. Of these, 721 (64.0%) were NAb-positive, indicating that, although most seropositive patients had generated antibodies, a large proportion failed to generate functionally effective antibodies capable of virus neutralization. Of patients tested for NAbs who were only IgA-positive, 41.7% (222/532) were NAb-positive. In contrast, 91.4% (427/467) of patients who were both IgA- and IgG-positive were NAb-positive, consistent with anti-viral antibody responses reflecting the process of affinity maturation^41,42^. A stronger correlation between IgG and NAbs versus IgA and NAbs was observed in a multivariate regression analysis (variables IgG [anti-S only] and IgA; *P* = 3.16e-14), at baseline (Supplemental Table 5).

We also evaluated the rate of seropositivity and NAb titers at baseline by risk factors for hospitalization, including age, sex, race, ethnicity, and viral load, in the Phase III portion of the trial (Supplemental Table 6). Subgroups defined by older age, male sex, being immunocompromised, and high viral titers were associated with a lower proportion of NAb positivity. In addition, for all subgroups except for older age, mean NAb titers were also lower. Differences were most notable for viral load, where a lower rate of seropositivity (~ 30% difference), a lower rate of NAb positivity (~ 60% difference) in seropositives, as well as lower mean NAb titers (lower by 1 log) in NAb positives, were observed. Further analyses showed an inverse relationship between the number of risk factors and NAb titers/percentage (Supplemental Table 6). In contrast, for subgroups characterized by other risk factors identified by the CDC^43^ (specifically, race and ethnicity), substantial differences in antibody profile were not clearly demonstrable. A summary of the number of risk factors and age groups by race and ethnicity is shown in Supplemental Table 7A and B, respectively.

### Association of baseline antibody profile with clinical outcomes and viral load

The presence of IgA and/or IgG antibodies at baseline was associated with differences in clinical outcomes. For seronegative patients who received placebo, 4.5% (39/860; 95% CI 3.2‒6.2) were hospitalized or died. For patients who received placebo and were IgA-positive only (i.e., positive on anti-S1 IgA, negative on both anti-S1 IgG and anti-N IgG) at baseline, the proportion of hospitalization/death was 3.9% (7/181; 95% CI 1.6‒7.8). However, for patients who received placebo and were both IgA- and IgG-positive (i.e., positive on anti-S1 IgA and positive on anti-S1 IgG or anti-N IgG), the proportion was 0.7% (1/167; 95% CI 0.01‒3.3), an 86.7% risk reduction compared with seronegative patients.

The association between antibody class and viral load was also examined at baseline. The viral loads (log10 copies/mL) for seronegative patients, those with IgA but not IgG, and those who had developed both IgA and IgG antibodies were 7.17 (SD: 1.46), 5.77 (SD: 1.59), and 4.36 (SD: 1.42), respectively, demonstrating a strong additive effect of IgG and IgA (40-fold decrease) relative to IgA alone on clearing the virus. Among the seropositive patients, the viral loads (log_10_ copies/mL) for NAb-positive and NAb-negative were 4.70 (SD: 1.44) and 6.81 (SD: 1.46), respectively.

### Evolution of symptoms with CAS + IMD

**Figure 1:**
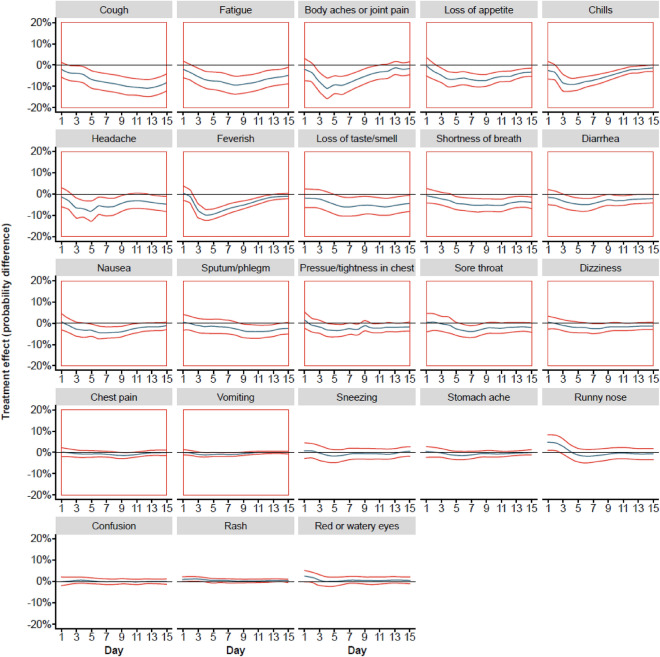
Treatment effect trajectories for patients enrolled in the adaptive Phase I/II/III CAS + IMD clinical trial. Treatment effect trajectories for each symptom of the Symptoms Evolution of COVID-19 instrument were obtained using a two-step approach (see Statistical Methodology for more details). The curve estimate is indicated by the blue line in the center, and 95% confidence bands are indicated by the red lines. The symptoms with significant treatment effects are highlighted in red boxes (upper bounds of the confidence bands below 0 for at least 2 consecutive days). The symptoms are ranked by area under the curve for the treatment effect trajectories relative to horizontal line y = 0.0%. *CAS* + *IMD* casirivimab + imdevimab, *COVID-19* coronavirus disease 2019.

The treatment effect on symptom trajectories estimated through the two-step approach is plotted in Fig. 1. The probability of 17 (of 23) individual symptoms (cough, fatigue, body aches, loss of appetite, chills, headache, feeling feverish, altered or loss of taste/smell, shortness of breath/difficulty breathing, diarrhea, nausea, sputum/phlegm, pressure/tightness in chest, sore throat, dizziness, chest pain, and vomiting) was significantly reduced with CAS + IMD treatment versus placebo (Fig. 1 and Supplemental Table 8). The six symptoms that showed no significant reduction were sneezing, stomachache, runny nose, confusion, rash, and red or watery eyes. Treatment effect trajectories were consistent across study cohorts (Supplemental Fig. 1). In addition, treatment effect trajectories were also estimated for an independent dataset including 1124 patients without risk factors for hospitalization (placebo: *n* = 397; CAS + IMD: *n* = 727) enrolled in the same clinical trial. Among these patients, CAS + IMD significantly reduced the probability of only two symptoms (loss of taste/smell and body aches) treatment versus placebo (Supplemental Fig. 2).

**Figure 2:**
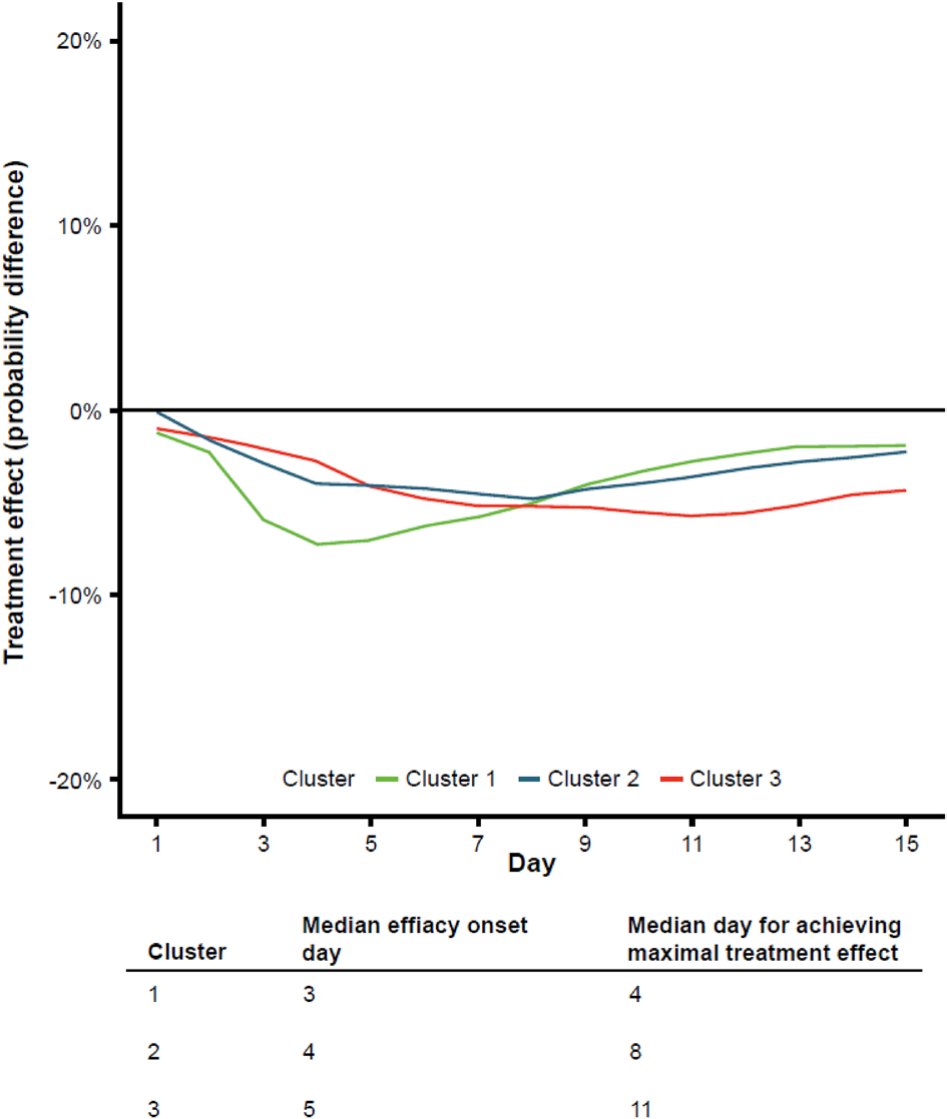
Treatment effect trajectories by symptom clusters. Hierarchical clustering was performed to group symptoms with similar treatment trajectories over time. Cluster 1 (early response) included headache, diarrhea, vomiting, feeling feverish, body aches, and chills; cluster 2 (mid-cycle of the 15-day observation) included pressure/tightness in chest, chest pain, loss of appetite, nausea, fatigue, and dizziness; and cluster 3 (delayed response) included cough, sputum/phlegm, sore throat, loss of taste/smell, and shortness of breath. An optimal number of clusters was determined using gap statistics.

Hierarchical clustering suggested that the 17 symptoms with significant treatment effects could be grouped into three clusters based on onset of treatment response (Supplemental Fig. 3A and B). Specifically, cluster 1 (early response) included headache, diarrhea, vomiting, feeling feverish, body aches, and chills; cluster 2 (mid-cycle of the 15-day observation) included pressure/tightness in chest, chest pain, loss of appetite, nausea, fatigue, and dizziness; and cluster 3 (delayed response) included cough, sputum/phlegm, sore throat, loss of taste/smell, and shortness of breath. For each cluster, the mean treatment effect trajectories are plotted in Fig. 2 and accompanied with median efficacy onset day (first day upper bound of 95% confidence band excluding 0%) and median day for achieving maximal treatment effect. As demonstrated in Fig. 2, CAS + IMD treatment had the most rapid and pronounced effects on symptoms in cluster 1. The median days of treatment onset were 3, 4, and 5 for clusters 1, 2, and 3, respectively, and the median days of maximal treatment effect were 4, 8, and 11, respectively.

### Association of baseline symptoms and evolution in CAS + IMD-treated patients with COVID-19–related hospitalization/death

As shown in Table 1, Cox regression analyses suggested that five symptoms (shortness of breath, cough, feeling feverish, fatigue, and loss of appetite) were significantly associated with incidence of hospitalization/death (*P* < 0.05 after adjustment for multiple comparisons by the Bonferroni method)^44^. For all five symptoms, a 1-point increase in severity score at baseline (e.g., mild vs moderate) was associated with a higher risk of hospitalization/death (cough: HR = 3.20, *P* < 0.001; fatigue: HR = 2.12, *P* < 0.001; feeling feverish: HR = 2.67, *P* < 0.001; loss of appetite: HR = 1.92, *P* < 0.001; shortness of breath: HR = 2.06, *P* = 0.001), whereas a reduction from baseline in severity score (e.g., moderate decreasing to mild) was associated with a lower risk of hospitalization/death (cough: HR = 0.31, *P* < 0.001; fatigue: HR = 0.51, *P* = 0.011; feeling feverish: HR = 0.47, *P* = 0.001; loss of appetite: HR = 0.56, *P* = 0.043; shortness of breath: HR = 0.34, *P* < 0.001). Furthermore, for each pair of the five symptoms, a composite symptom variable was derived by taking an average of the two symptoms. Similar Cox regression analyses were performed on these composite symptom variables. As shown in Supplemental Table 9, the pair of cough and feeling feverish had the greatest association with hospitalization/death. For every one-point increase in severity at baseline (e.g., from mild-to-moderate) for this pair of symptoms, the risk of hospitalization/death increased 4.54-fold, and for every one-point decrease in severity from baseline (e.g., from moderate-to-mild), the risk of hospitalization/death decreased 0.78-fold. All five symptoms improved significantly with CAS + IMD treatment.

**Table 1:** Cox regression analysis of the relationship between severity of individual COVID-19 symptoms and hospitalization/death.

Symptom	Baseline (per 1-point increase, e.g., mild-to-moderate)		Change from baseline (per 1-point decrease, e.g., moderate-to-mild)	
	HR (95% CI)	*P*-value	HR (95% CI)	*P*-value
Shortness of breath*	2.06 (1.45–2.93)	0.001	0.34 (0.25–0.45)	< 0.001
Cough*	3.20 (2.25–4.53)	< 0.001	0.31 (0.23–0.43)	< 0.001
Feeling feverish*	2.67 (1.97–3.61)	< 0.001	0.47 (0.33–0.68)	0.001
Fatigue*	2.12 (1.58–2.85)	< 0.001	0.51 (0.35–0.74)	0.011
Loss of appetite*	1.92 (1.48–2.49)	< 0.001	0.56 (0.39–0.81)	0.043
Nausea	1.50 (1.03–2.16)	0.759	0.53 (0.36–0.80)	0.050
Chills	2.06 (1.53–2.78)	< 0.001	0.56 (0.38–0.81)	0.058
Dizziness	1.85 (1.33–2.56)	0.005	0.56 (0.35–0.91)	0.423
Sore throat	1.11 (0.74–1.68)	1.000	0.67 (0.44–1.03)	1.000
Vomiting	0.95 (0.34–2.65)	1.000	0.70 (0.32–1.56)	1.000
Diarrhea	1.28 (0.92–1.78)	1.000	0.82 (0.51–1.32)	1.000
Headache	1.35 (0.95–1.92)	1.000	0.81 (0.56–1.17)	1.000
Red or watery eyes	1.30 (0.82–2.08)	1.000	1.11 (0.69–1.80)	1.000
Body aches or joint pain	1.48 (1.10–1.99)	0.229	0.76 (0.54–1.07)	1.000
Loss of taste/smell	0.94 (0.71–1.23)	1.000	1.02 (0.74–1.39)	1.000
Confusion	1.64 (0.96–2.80)	1.000	0.57 (0.30–1.09)	1.000
Pressure/tightness in chest	1.82 (1.30–2.54)	0.011	0.66 (0.36–1.21)	1.000
Chest pain	2.29 (1.53–3.41)	0.001	0.84 (0.51–1.38)	1.000
Stomachache	1.26 (0.78–2.04)	1.000	0.67 (0.37–1.23)	1.000
Rash	1.73 (0.74–4.09)	1.000	0.67 (0.27–1.64)	1.000
Sneezing	0.77 (0.42–1.42)	1.000	1.13 (0.52–2.43)	1.000
Runny nose	1.07 (0.70–1.65)	1.000	0.75 (0.47–1.19)	1.000
Sputum/phlegm	1.67 (1.19–2.34)	0.064	0.74 (0.48–1.13)	1.000

A time-varying Cox regression model was fit to each symptom of the Symptoms Evolution of COVID-19 instrument individually, with time to hospitalization/death as the outcome with the following covariates: baseline symptom score (none = 0, mild = 1, moderate = 2, severe = 3), daily symptom change from baseline, age, sex, BMI, treatment indicator, and baseline viral load (in log_10_ scale). Reported *P*-values were adjusted by the Bonferroni approach for baseline and change from baseline, respectively.

*P < 0.05 for both baseline and change from baseline. *BMI* body mass index, *COVID-19* coronavirus disease 2019.

### Symptom resolution with CAS + IMD in key subgroups

**Figure 3:**
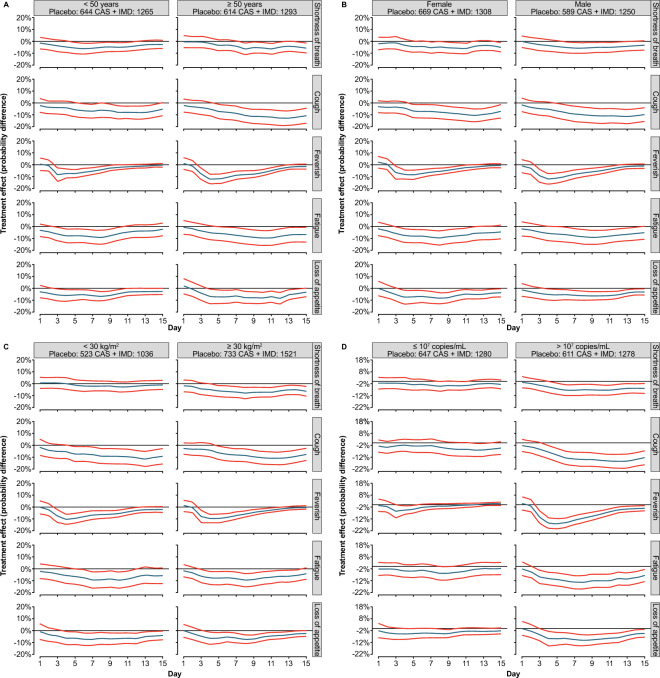
Treatment effect trajectories based on baseline patient characteristics and baseline viral load. (**A**) Age, (**B**), sex, (**C**) obesity, and (**D**) viral load. (**A–D**) Treatment effect trajectories for each symptom of the Symptoms Evolution of COVID-19 instrument were obtained for patients treated with placebo or CAS + IMD using a two-step approach and analyzed by age, sex, BMI, and baseline viral load (see Statistical Methodology for more details). Curve estimates are indicated by the blue lines in the center, and 95% confidence bands are indicated by the red lines. *BMI* body mass index, *CAS* + *IMD* casirivimab + imdevimab, *COVID-19* coronavirus disease 2019.

Subgroup analyses focusing on the five symptoms most associated with hospitalization/death based on baseline demographic factors and baseline viral load are presented in Fig. 3. Shifts in symptom trajectories suggested greater treatment effects (active vs placebo) for patients ≥ 50 years of age than those < 50 years of age, especially for the symptoms of cough and feeling feverish (Fig. 3A). Treatment effects were comparable between female and male patients (Fig. 3B). Patients with obesity received greater treatment benefit, especially in the symptom shortness of breath, than those without obesity (Fig. 3C). As shown in Fig. 3D, greater treatment effect was also observed for all five core symptoms in patients with high baseline viral load (> 10^7^ copies/mL) than in those with low baseline viral load (≤ 10^7^ copies/mL). The results of these analyses were consistent in showing that patients at high risk for hospitalization achieved quicker resolution of symptoms after receiving treatment with CAS + IMD compared with placebo.

### Symptom resolution with CAS + IMD according to antibody profile

**Figure 4:**
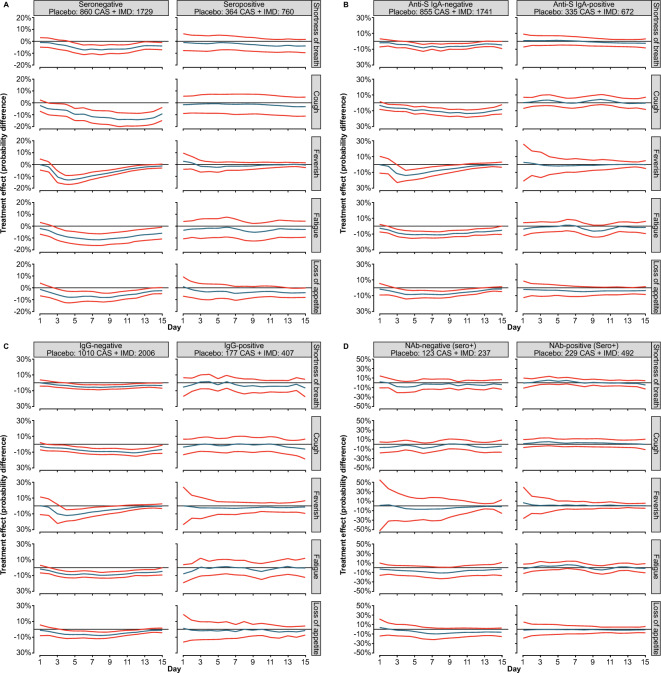
Treatment effect trajectories based on baseline antibody profile. (**A**) serology, (**B**) IgA, (**C**) IgG, and (**D**) Nabs. (**A–D**) Treatment effect trajectories for each symptom of the Symptoms Evolution of COVID-19 instrument were obtained using a two-step approach and analyzed by baseline serology status and baseline Ig status (see Statistical Methodology for more details). Antibody class development was considered (seronegative versus seropositive, IgA-negative versus IgA-positive, IgG-negative versus IgG-positive, and NAb-negative versus NAb-positive) in the analysis of treatment effect trajectories by baseline Ig status. Curve estimates are indicated by the blue lines in the center, and 95% confidence bands are indicated by the red lines. *CAS* + *IMD* casirivimab + imdevimab, *COVID-19* coronavirus disease 2019, *Ig* immunoglobulin, *NAb* neutralizing antibody.

The results of subgroup analyses based on Ig status at baseline are presented in Fig. 4. The analyses showed that treatment effects were greater in patients who lacked antibodies (either IgA or IgG) at baseline (i.e., seronegative) compared with patients with antibodies at baseline (i.e., seropositive) (Fig. 4A–C).

Further analyses of symptoms resolution focused on patients who were seropositive at baseline to explore potential differences in response to treatment within this subgroup. Analysis by the presence/absence of NAbs at baseline demonstrated a significant treatment benefit of CAS + IMD in patients who lacked NAbs, while there was no difference in those who were NAb-positive (Fig. 4D).

In analyses of patients receiving placebo only, the presence of IgA at baseline impacted symptom evolution for cough and possibly fatigue, whereas the presence of IgG had a favorable effect on fever and possibly fatigue (Supplemental Fig. 4A and B). For patients with baseline NAbs versus those without, the evolutions of the five key symptoms were comparable (Supplemental Fig. 4).

**Figure 5:**
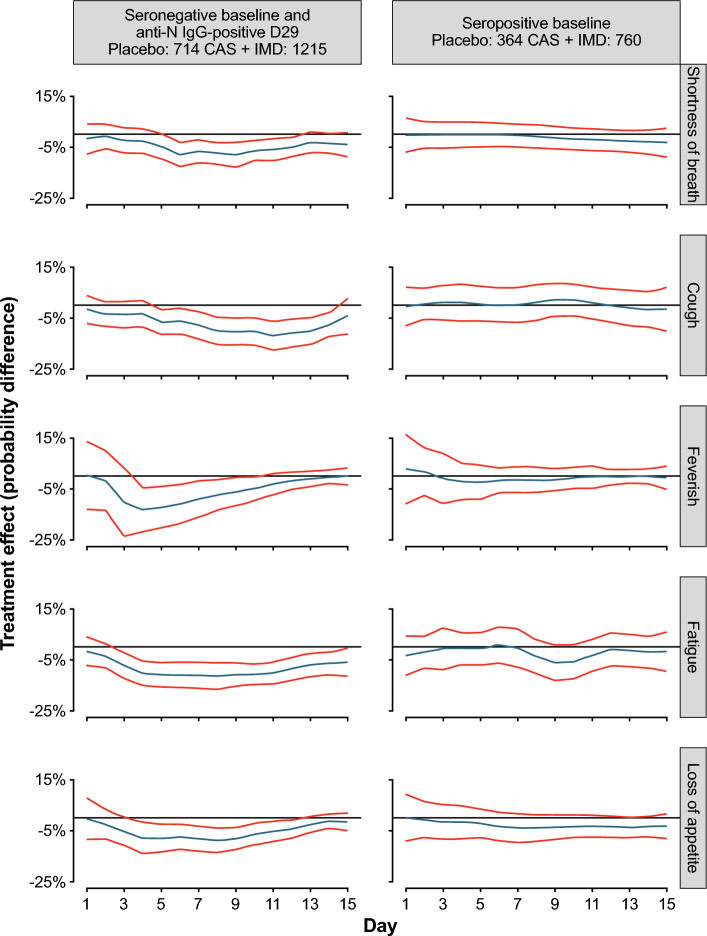
Treatment effect trajectories based on seropositivity at baseline and seroconversion by day 29. Treatment effect trajectories for each symptom of the Symptoms Evolution of COVID-19 instrument were obtained using a two-step approach and analyzed by baseline serology status and seroconversion (see Statistical Methodology for more details). Baseline seronegative patients who seroconverted by day 29 versus seropositive patients at baseline were considered in the analysis of treatment effect trajectories. Curve estimates are indicated by the blue lines in the center, and 95% confidence bands are indicated by the red lines. *anti-N *anti-nucleocapsid, *CAS* + *IMD* casirivimab + imdevimab, *COVID-19* coronavirus disease 2019, *Ig* immunoglobulin.

We also examined the effects of CAS + IMD on symptom evolution in patients who demonstrated late seroconversion (i.e., seronegative at baseline but seropositive for IgG anti-N by day 29; Fig. 5). Results showed a benefit of treatment vs placebo across all five symptoms in late-seroconverted patients. Furthermore, patients receiving no treatment, but who had seroconverted late, demonstrated little difference in symptom evolution compared with those who remained non-converted (i.e., seronegative at day 29) (Supplemental Fig. 4D).

## Discussion

This secondary analysis reports the results from a clinical trial designed to determine the effect of CAS + IMD treatment in outpatients with acute COVID-19^19^, focusing on high-risk symptomatic patients to examine the effect of CAS + IMD on individual symptom resolution and to characterize the relationship between treatment effect and risk factors associated with severe COVID-19. Furthermore, our analysis assessed the effect of endogenous anti–SARS-CoV-2 antibodies present at randomization on the efficacy of treatment with CAS + IMD. While a few publications have described acute COVID-19^14,45^, none have assessed symptom resolution, relationship to antibody profile, or association with outcomes^13,14,17,20,38,45^.

We delineated the effect of treatment on individual symptoms that individually and collectively are important to consider when evaluating response to treatment in a clinical setting, showing that three symptom clusters were most responsive to CAS + IMD, as demonstrated by correlation analysis and hierarchical clustering. These symptom complexes appeared and resolved in different time periods after acute presentation.

We found that the reduction in symptoms with CAS + IMD treatment compared to placebo varied according to the presence or absence of established risk factors associated with severe COVID-19 outcomes (e.g., age, obesity, and baseline viral load)^14,17^. Symptom improvement following treatment with CAS + IMD was generally more notable in patients ≥ 50 years of age, those with high BMI, or those with high baseline viral load than in younger patients, those with low BMI, or those with low baseline viral load, highlighting the need to provide specific, virus-neutralizing antibodies to optimally resolve symptoms in individuals at the highest risk of COVID-19 hospitalization^14^. Our analyses also suggested that patients with risk factors for severe COVID-19 who generally exhibited lower rates of seropositivity and neutralizing antibodies exhibited a much greater treatment benefit across the spectrum of symptoms than those without these risk factors (Fig. 1 and Supplemental Fig. 1).

Five symptoms (shortness of breath, cough, feeling feverish, fatigue, and loss of appetite) were strongly associated with occurrence of hospitalization/death. This confirms and extends findings of other studies that used independent data sets^14,16^. Of these five symptoms, severity of feeling feverish may be the most important predictor of poor outcomes, reflecting the intensity of infection and the ensuing cytokine storm with attendant effects on organ function, as well as on sense of well-being^16^. Loss of appetite may have analogous significance, given that it is strongly correlated with the presence of fever symptoms^46^.

A feature of the statistical analysis pursued here is the application of a bootstrapping method. The results suggest the bootstrapping subsamples for the symptom of “feeling feverish” exhibited a wider variance in the treatment effect (i.e., probability difference) in some sub-groups compared with the other symptoms, noting that in the overall seronegative population, CAS + IMD provided clear evidence of resolution of the symptom of “feeling feverish”.

Adaptive immunity data from vaccine responses^33^ demonstrated that NAbs were highly predictive of immune protection, with even modest levels providing protection against the most severe outcomes. Their impact on the resolution of symptoms, however, is less clear, since the natural production of antibodies may be associated with the immune response to the virus. Moreover, the timing of NAb production has been found to correlate with fatal COVID-19 outcomes, with NAbs generated before day 14 of symptom onset being strongly associated with recovery^34^. Therefore, our findings confirm and extend the relationship between specific symptoms and the timing and quality of antibody production and outcomes in the setting of treatment with SARS-CoV-2-specific, highly neutralizing antibodies.

Measurement of anti–SARS-CoV-2 antibodies also provided an opportunity to assess the effect of baseline antibody responses on time to symptom resolution. Results demonstrated clear differences in treatment response, with a greater magnitude of benefit in patients who were seronegative (i.e., negative on all anti-S1 IgA, anti-S1 IgG, and anti-N IgG antibodies at baseline) versus those who were seropositive at baseline across all five symptoms. A treatment benefit was also observed in patients who seroconverted by day 29 versus those who were seropositive at baseline, underscoring the importance of early administration of CAS + IMD in rescuing patients lacking early serologic responses to SARS-CoV-2.

Our analyses also further clarify the role of Ig class and resolution of COVID-19 infection. The treatment benefits in the IgA-positive group were generally similar to the IgG-positive group, suggesting that IgA generated very early on contributes to virus neutralization, as previously suggested by others^47^. However, we demonstrate that IgG may be a more critical immune element to protect patients from progressing to a serious clinical outcome (e.g., hospitalization/death), since the appearance of the IgG antibody in a maturing immune response maximizes neutralizing capacity. The effect of CAS + IMD, highly neutralizing IgG antibodies directed at the receptor-binding domain, confirms the importance of SARS-CoV-2–directed IgG in improving outcomes.

These findings also confirm and extend our understanding of the role of virus-neutralizing antibodies in the resolution of acute COVID-19 infection. The failure to generate NAbs early in the disease course has been shown to be a risk factor for mortality in natural infection^34^. Similarly, the generation of NAbs in response to vaccination is a strong predictor of preventing infection^33^. In our study, seropositivity, defined by the presence of antibodies to SARS-CoV-2 directed against spike protein or nucleocapsid, significantly correlated with the presence of NAbs, given that 74% of seropositive patients had measurable NAbs. However, approximately 25% of the seropositive population did not produce antibodies that could neutralize the virus, thus providing a window for treatment benefit even in those who are seropositive on presentation, as we have previously shown for hospitalized patients^48^.

While there is a growing body of evidence of the roles T cells and non-neutralizing antibodies play in immunity against COVID-19, this largely applies to their adjunctive contributions to the overwhelmingly dominant role of neutralizing antibodies in clearing acute viral disease or in the case of the former, prophylaxis against reinfection or new infections after vaccinations when antibody responses have waned^49–51^. Notably, this study focused on acute COVID-19 infection, symptoms and outcomes, and the role antibodies play in its resolution. In our study, we demonstrate that highly specific monoclonal antibody treatment can be an important tool in the management of patients who cannot mount early and specific antibody responses to COVID-19. Current data suggest no substantive interference in the development of the endogenous immune response after treatment with the highly specific monoclonal antibody cocktail studied here, CAS + IMD^52,53^. Future research is needed to understand the contribution of both T cells and non-neutralizing antibody responses in the evolution of de novo, acute COVID-19 infection.

Although endogenous NAbs were not specifically tested for antibody class, a relatively low percentage (~ 42%) of IgA-positive, IgG-negative patients had detectable NAb activity at baseline, whereas almost all dual IgA- and IgG-positive patients demonstrated neutralizing capacity. This highlights the importance of affinity maturation of antibody response reflected by emergence of detectable virus-specific IgG production, leading to highly effective anti-virus NAbs that are critical for symptom resolution and clinical outcomes. Although NAbs assessed in serum might underestimate the role of mucosal IgA with the potential to neutralize virus locally^26^, the association of improved outcomes in individuals with a more mature antibody response, and key symptom resolution and improved outcomes with IgG antibodies provided with administration of CAS + IMD, demonstrate the critical role of IgG in the resolution of severe COVID-19 illness^22,32^.

In summary, high severity and long duration of a set of symptoms identified here were found to be associated with poor COVID-19 outcomes. The effects of CAS + IMD treatment on symptoms and the role of antibody class revealed new relationships and extend previous reports of the benefit of this therapeutic with respect to associated morbidity and mortality, most notably in patients lacking an anti–SARS-CoV-2 serologic response at baseline^38^. Furthermore, this study provides the first evidence of the critical role of endogenous SARS-CoV-2–specific IgG produced early in the disease in resolving specific COVID-19 symptoms. This work also supports the role of treatment with highly neutralizing monoclonal IgG antibodies at the earliest signs of disease to accelerate symptom resolution. Whether a potent neutralizing IgG combination of CAS + IMD can counteract low-titer antibody responses at baseline that may be misdirected, or represent cross-reactive antibodies from previous viral infections that are SARS-CoV-2 viral enhancing^54^, was not studied, but deserves further attention. Finally, and most importantly, in patients with risk factors for severe COVID-19 or in those who have inadequate early serologic responses, more rapid resolution of a set of symptoms following CAS + IMD treatment signaled reduction of risk of the worst outcomes of hospitalization and death.

## Methods

### Study population

This study included symptomatic outpatients with SARS-CoV-2 infection who were at high risk of severe COVID-19 (e.g., ≥ 50 years of age, BMI ≥ 30 kg/m^2^) and enrolled in the COV-2067 clinical trial (NCT04425629). Eligible patients were ≥ 18 years of age and had a positive SARS-CoV-2 diagnostic test (RT-qPCR or antigen) from a sample collected ≤ 72 h prior to randomization. In addition, eligible patients had symptoms consistent with COVID-19 with onset ≤ 7 days before randomization and ≥ 93% oxygen saturation on room air^39^.

### Study treatments and design

Data used in this study were derived from an adaptive, seamless, randomized, double-blind, placebo-controlled Phase I/II/III clinical trial that evaluated the efficacy, safety, and tolerability of CAS + IMD in outpatients with COVID-19. The trial enrolled patients with varying baseline characteristics over time and varied the doses administered in an adaptive fashion^38,39^. Patients in this study were enrolled prior to the emergence of Omicron-lineage variants.

Briefly, patients in the Phase I/II portion of the trial (*n* = 799) were randomized 1:1:1 to placebo or to a single intravenous dose of CAS + IMD 2400 mg or 8000 mg. The Phase III portion was amended such that subsequent patients were randomized 1:1:1 to placebo, CAS + IMD 1200 mg, or CAS + IMD 2400 mg, and had ≥ 1 risk factor for developing severe COVID-19^38,39^.

The total duration of the symptom assessment period was 28 days (days 1–29), although here we present data up to day 15. Throughout the study, patients used an electronic diary to provide daily self-reported information about COVID-19 symptoms^19^.

### Clinical outcomes

The primary endpoint for the Phase III portion of the trial was the proportion of patients with ≥ 1 COVID-19–related hospitalization or all-cause death through day 29.

### Measurement of SARS-CoV-2 symptoms

Symptom data were collected using the Symptoms Evolution of COVID-19 (SE-C19^©^), an electronic diary that was developed by Regeneron Pharmaceuticals, Inc. based on available data on symptoms of COVID-19 to assess COVID-19 symptom evolution over time^19^. In SE-C19, patients were presented with a list of 23 symptoms (Supplemental Table 10) and were asked to confirm which they had experienced, as well as to rate the severity of experienced symptoms at their worst moment in the last 24 h (none [0], mild [1], moderate [2], or severe [3]).

### Measurement of anti–SARS-CoV-2 Igs (IgG-S, IgA-S, IgG-N, and NAbs)

Serum samples were collected prior to treatment for the determination of composite anti–SARS-CoV-2 serostatus, as well as the presence of Ig/antigen-specific anti–SARS-CoV-2 antibody responses and NAbs, as previously described^39,48^. Composite serostatus was determined for all patients using the following validated anti–SARS-CoV-2 serology tests: EuroImmun (Lübeck, Germany) anti–SARS-CoV-2 ELISA IgA (which measures the anti-S1 domain of spike protein IgA antibodies), EuroImmun anti–SARS-CoV-2 ELISA IgG (which measures anti-S1 IgG antibodies), and Abbott (Chicago, USA) SARS-CoV-2 IgG Architect (which measures anti-N protein IgG antibodies). Note that the CAS + IMD antibody combination is not detected by the anti-N IgG serology test. At baseline, individuals were deemed seronegative when all three available test results were negative, or seropositive when any available test result was positive. To determine seroconversion at day 29, individuals were considered positive using results from the Abbott SARS-CoV-2 IgG Architect (which measures anti-N protein IgG antibodies) serology test only. The status of “other” was used for any patients who did not have any test results available (e.g., sample missing) or who had ≥ 1 borderline test result in the absence of any positive test results. Neutralizing titer assays (NTAs) were also performed on samples from 1124 baseline-seropositive patients. Serum samples were tested using the IMMUNO-COV assay^55^, a recombinant vesicular stomatitis virus assay that quantitates SARS-CoV-2 NAbs. Performance characteristics of the three anti–SARS-CoV-2 serology assays and the NTA assay demonstrated high sensitivity and specificity for the detection of prior SARS-CoV-2 infection, either as single assays or as composite serostatus (Hooper et al. 2023, manuscript in preparation).

### Analysis population and symptom data analysis period

The analysis population for this study included all randomized patients with positive RT-qPCR in nasopharyngeal (NP) swab samples at randomization and with ≥ 1 risk factor for hospitalization (Supplemental Table 1). In addition, only patients who had symptom data at baseline (day 1) and ≥ 1 follow-up day between days 2 and 15 were included in the analysis. Symptom data up to day 15 were included in the analysis because most symptoms (except for cough, fatigue, loss of taste/smell, and headache) resolved by day 16 (Supplemental Table 11). In this analysis, with a focus on understanding the overall evolution of symptoms and their relationship to CAS + IMD treatment, data from patients from all phases from the study were pooled unless specified otherwise. Furthermore, all CAS + IMD doses (1.2, 2.4, and 8.0 g) were pooled because the clinical efficacy of these doses was similar^38^.

### Baseline characteristics

The following baseline characteristics were summarized for patients in each treatment arm: age, sex, ethnicity, race, weight, height, BMI, obesity status, baseline viral load measured by NP swab samples, rating of symptoms present at baseline, and serology status indicating prior exposure to SARS-CoV-2 infection. In relation to serology status, anti–SARS-CoV-2 NAb status and anti–SARS-CoV-2 IgA/IgG status were evaluated.

### Statistical methodology

To understand how treatment with CAS + IMD affects improvement of clinical symptoms over time, a two-step approach was applied to the longitudinal symptom data^56^. In step one, for each symptom at each day, a logistic regression model was fitted to the presence/absence of a symptom on the following variables: treatment (0 = placebo/1 = CAS + IMD), age, sex, BMI, and baseline viral load (log_10_ scale). ${\widehat{p}}_{i,REGEN-COV}^{d}$ and ${\widehat{p}}_{i,placebo}^{d}$ denoted the least-squares means estimates of probabilities for presence of the ${i}^{th}$ symptom ($i=$ feeling feverish, chills, loss of taste/smell, etc.) at day $d$ ($d=\mathrm{1,2},\dots ,15)$ based on the logistic regression model. In step two, an estimate of treatment effect (quantified by the probability difference between treatment arms, i.e., ${\Delta }_{i}^{d}={p}_{i,REGEN-COV}^{d}$ – ${p}_{i,placebo}^{d}$) over time was obtained for each symptom by smoothing the raw estimates (${\widehat{\Delta }}_{i}^{d}{^\prime}s)$ from the first step based on a smoothing method proposed for coefficients for logistic regression models^56^. Ninety-five percent global confidence bands for the treatment effect functions were constructed based on the bootstrap method (300 replications), which enabled the identification of symptoms with significant treatment benefit as well as the period during which symptoms were significantly reduced by CAS + IMD. Symptoms with significant treatment effects were defined as those with the associated upper bound of the 95% confidence band being below 0 for ≥ 2 consecutive days.

Hierarchical clustering was performed to group symptoms with similar treatment trajectories over time. An optimal number of clusters was determined using gap statistics^57^. For each pair of symptoms, similarity between treatment trajectories was quantified by one—Pearson correlation.

Cox regression modeling was used to identify symptoms associated with a poor clinical outcome (i.e., hospitalization/death). For each symptom, the Cox regression model was fit using time to hospitalization/death as the outcome with the following covariates: baseline symptom score, daily symptom score change from baseline (time-varying), treatment indicator, age, sex, baseline BMI, and baseline viral load (in log_10_ scale).

Subgroup analyses were performed using the same two-step approach as described above for the following variables: age (< 50 years vs ≥ 50 years); sex; BMI (< 30 kg/m^2^ vs ≥ 30 kg/m^2^); baseline viral load (≤ 10^7^ vs > 10^7^ copies/mL); baseline serology status (seronegative vs seropositive); and Ig status, which took antibody class development into consideration (seronegative vs IgA-positive, and IgG-negative vs IgA-positive and IgG-positive). For all subgroup analyses involving baseline serology/Ig, patients with an outcome of IgA and IgG (anti-S) “borderline” were included in the positive subgroup, since quantitative retesting of a subset of these samples demonstrated that they were much more likely to be positive than negative. Spearman rank correlation coefficients were used to examine the relationships between different antibody classes.

All statistical analyses were done in R version 4.1.2 with the following packages:

- Emmeans (version 1.7.2) was used to generate the least-squares estimates from the logistic model^58^.
- Survival (version 3.2.13) was used to generate the HRs via the Cox model^59^.

### Ethics

The trial was conducted in accordance with the principles of the Declaration of Helsinki, International Council for Harmonisation Good Clinical Practice guidelines, and applicable regulatory requirements. All patients provided written informed consent before participating in the trial. The local institutional review board or ethics committee at each study center oversaw trial conduct and documentation. Ethics approval was obtained from the following ethics review boards: WCG IRB, Puyallup, WA (IRB00000533); Medicasur, Mexico City, Mexico (20CI09012015, CB9012009 & 20CI09012015); Hospital La Mision S.A. de C.V, Monterrey, Mexico (IRB00011076, COBBIOTICA-19-CEI-008-20160729 & 20CI09012015); Providence St Joseph's Health, Renton, WA (STUDY2020000419 & STUDY2020000465); Lifespan—Rhode Island Hospital, Providence, RI (CMTT/PROJ no: 213620); Research Compliance Office, Palo Alto, CA (IRB 5 Registration4593/Eprotocol: 57728); Advarra IRB, Columbia, MD (MOD009333300).

### Supplementary Information

Supplementary Information.

## Data Availability

Qualified researchers can submit a proposal for access to individual patient or aggregate level data from a Regeneron-sponsored clinical trial through Vivli (https://vivli.org/).
