# Supplementary material for: Casirivimab + imdevimab accelerates symptom resolution linked to improved COVID-19 outcomes across susceptible antibody and risk profiles
Source: Sci Rep. 2023 Aug 7;13:12784. doi: 10.1038/s41598-023-39681-7 (PMC10406852; doi:10.1038/s41598-023-39681-7)
Supplement: Supplementary file 1 — Supplementary Information. [file 41598_2023_39681_MOESM1_ESM.docx]

# Supplementary Material

Supplemental Table 1. Risk factors for hospitalization

| **Category** |
| --- |
| ≥ 50 years of age |
| Obesity, defined as body mass index ≥ 30 kg/m^2^ |
| Cardiovascular disease (e.g., hypertension, prior myocardial infarction, stroke) |
| Chronic lung disease (e.g., asthma, chronic obstructive pulmonary disease) |
| Chronic metabolic disease (e.g., diabetes) |
| Chronic kidney disease, including those on dialysis |
| Chronic liver disease |
| Immunosuppressed state, due to taking an immunosuppressant drug or having an immunological disease resulting in immunosuppression, based on the investigator’s assessment (e.g., cancer, cancer treatment, bone marrow or organ transplantation, or immune deficiencies) |

Supplemental Table 2. Patient characteristics at baseline

**A** Summary of demographics and risk factors

|  | **Placebo (*n* = 1258)** | **CAS + IMD *(n* = 2558)** | **Total (*N* = 3816)** |
| --- | --- | --- | --- |
| Age, years |  |  |  |
| Mean (SD) | 47.2 (14.1) | 48.2 (14.6) | 47.9 (14.4) |
| Median | 49 | 50 | 49 |
| Q1 : Q3 | 37 : 57 | 38 : 58 | 37 : 58 |
| Min : Max | 18 : 92 | 18 : 96 | 18 : 96 |
| Age group (years), *n* (%) |  |  |  |
| 18–44 | 513 (40.6) | 996 (38.9) | 1509 (39.4) |
| 45–64 | 628 (49.7) | 1224 (47.8) | 1852 (48.4) |
| 65–84 | 113 (8.9) | 325 (12.7) | 438 (11.4) |
| ≥ 85 | 4 (0.3) | 13 (0.5) | 17 (0.4) |
| Sex, *n* (%) |  |  |  |
| Female | 669 (53.2) | 1308 (51.1) | 1977 (51.8) |
| Male | 589 (46.8) | 1250 (48.9) | 1839 (48.2) |
| Ethnicity, *n* (%) |  |  |  |
| Hispanic or Latino | 462 (36.7) | 926 (36.2) | 1388 (36.4) |
| Not Hispanic or Latino | 789 (62.7) | 1611 (63.0) | 2400 (62.9) |
| Not reported | 7 (0.6) | 21 (0.8) | 28 (0.7) |
| Race, *n* (%) |  |  |  |
| White | 1075 (85.5) | 2181 (85.3) | 3256 (85.3) |
| Black or African American | 65 (5.2) | 134 (5.2) | 199 (5.2) |
| Asian | 48 (3.8) | 91 (3.6%) | 139 (3.6) |
| American Indian or Alaska Native | 7 (0.6) | 30 (1.2) | 37 (1.0) |
| Unknown | 35 (2.8) | 69 (2.7) | 104 (2.7) |
| Not reported | 27 (2.1) | 47 (1.8) | 74 (1.9) |
| Weight, kg |  |  |  |
| Mean (SD) | 90.1 (21.7) | 91.1 (22.4) | 90.8 (22.2) |
| Median | 88.4 | 88.5 | 88.5 |
| Q1 : Q3 | 74.9 : 103.22 | 75.8 : 104 | 75.3 : 103.8 |
| Min : Max | 43.1 : 198.7 | 43 : 228.6 | 43 : 228.6 |
| Height, cm |  |  |  |
| Mean (SD) | 169.2 (11.2) | 169.6 (10.8) | 169.5 (11) |
| Median | 168 | 170 | 170 |
| Q1 : Q3 | 161 : 177.8 | 162.5 : 203.2 | 162 : 177.8 |
| Min : Max | 114 : 218.4 | 123 : 203.2 | 114 : 218.4 |
| BMI, kg/m^2^ |  |  |  |
| Mean (SD) | 31.4 (6.5) | 31.6 (6.9) | 31.5 (6.8) |
| Median | 31 | 30.9 | 30.9 |
| Q1 : Q3 | 26.7 : 34.9 | 27.1 : 34.8 | 27 : 34.8 |
| Min : Max | 16.2 : 62 | 15.9 : 67.3 | 15.9 : 67.3 |
| Obesity, *n* (%) |  |  |  |
| BMI < 30 kg/m^2^ | 523 (41.6) | 1036 (40.5) | 1559 (40.9) |
| BMI ≥ 30 kg/m^2^ | 733 (58.3) | 1521 (59.5) | 2254 (59.1) |
| Missing | 2 (0.2) | 2 (0.1) | 4 (0.1) |
| Risk factor, *n* (%) |  |  |  |
| Obesity, defined as  BMI ≥ 30 kg/m^2^ | 733 (58.3) | 1521 (59.5) | 2254 (59.1) |
| Age ≥ 50 years | 614 (48.8) | 1293 (50.5) | 1907 (50.0) |
| Cardiovascular disease, including hypertension | 436 (34.7) | 908 (35.5) | 1344 (35.2) |
| Chronic lung disease, including asthma | 196 (15.6) | 425 (16.6) | 621 (16.3) |
| Chronic metabolic disease, including diabetes | 197 (15.7) | 366 (14.3) | 563 (14.8) |
| Immunocompromised | 27 (2.1) | 77 (3.0) | 104 (2.7) |
| Chronic kidney disease, including those on dialysis | 9 (0.7) | 37 (1.4) | 46 (1.2) |
| Chronic liver disease | 9 (0.7) | 27 (1.1) | 36 (0.9) |
| Taking immunosuppressants | 9 (0.7) | 18 (0.7) | 27 (0.7) |

BMI, body mass index; SD, standard deviation.

**B** Outcome of hospitalization and/or death

|  | **Placebo (*n* = 1258)** | **CAS + IMD (*n* = 2558)** | **Total (*N* = 3816)** |
| --- | --- | --- | --- |
| Hospitalization and/or all-cause mortality, *n* (%) |  |  |  |
| No | 1210 (96.2) | 2538 (99.2) | 3748 (98.2) |
| Yes | 48 (3.8) | 20 (0.8) | 68 (1.8) |
| Hospitalization, *n* (%) | 48 (3.8) | 20 (0.8) | 68 (1.8) |
| All-cause mortality, *n* (%) | 3 (0.2) | 1 (<0.1) | 4 (0.1) |

**C** Symptoms summary

**i** Summary by treatment arm

| ***n* (%)** | **Placebo *(n* = 1258)** | **CAS + IMD (*n* = 2558)** | **Total (*N* = 3816)** |
| --- | --- | --- | --- |
| Cough | 911 (72.4) | 1821 (71.2) | 2732 (71.6) |
| Fatigue | 828 (65.8) | 1643 (64.2) | 2471 (64.8) |
| Headache | 783 (62.2) | 1552 (60.7) | 2335 (61.2) |
| Body aches or joint pain | 689 (54.8) | 1354 (52.9) | 2043 (53.5) |
| Loss of taste/smell | 589 (46.8) | 1123 (43.9) | 1712 (44.9) |
| Chills | 471 (37.4) | 898 (35.1) | 1369 (35.9) |
| Runny nose | 446 (35.5) | 987 (38.6) | 1433 (37.6) |
| Sore throat | 431 (34.3) | 866 (33.9) | 1297 (34.0) |
| Loss of appetite | 419 (33.3) | 851 (33.3) | 1270 (33.3) |
| Feeling feverish | 380 (30.2) | 775 (30.3) | 1155 (30.3) |
| Sneezing | 355 (28.2) | 720 (28.1) | 1075 (28.2) |
| Shortness of breath | 306 (24.3) | 601 (23.5) | 907 (23.8) |
| Diarrhea | 282 (22.4) | 538 (21.0) | 820 (21.5) |
| Sputum/phlegm | 278 (22.1) | 580 (22.7) | 858 (22.5) |
| Nausea | 266 (21.1) | 568 (22.2) | 834 (21.9) |
| Red or watery eyes | 228 (18.1) | 510 (19.9) | 738 (19.3) |
| Dizziness | 219 (17.4) | 450 (17.6) | 669 (17.5) |
| Pressure/tightness in chest | 199 (15.8) | 436 (17.0) | 635 (16.6) |
| Stomachache | 162 (12.9) | 312 (12.2) | 474 (12.) |
| Chest pain | 92 (7.3) | 200 (7.8) | 292 (7.7) |
| Confusion | 89 (7.1) | 176 (6.9) | 265 (6.9) |
| Vomiting | 50 (4.0) | 89 (3.5) | 139 (3.6) |
| Rash | 20 (1.6) | 54 (2.1) | 74 (1.9) |

**ii** Summary by treatment arm and symptom severity

| **Severity, *n* (%)** | **Placebo (*n* = 1258)** | | | | **CAS + IMD (*n* = 2558)** | | | | **Total (*N* = 3816)** | | | |
| --- | --- | --- | --- | --- | --- | --- | --- | --- | --- | --- | --- | --- |
|  | **None** | **Mild** | **Moderate** | **Severe** | **None** | **Mild** | **Moderate** | **Severe** | **None** | **Mild** | **Moderate** | **Severe** |
| Cough | 347  (27.6) | 490  (39.0) | 352  (28.0) | 69  (5.5) | 737  (28.8) | 1019 (39.8) | 636  (24.9) | 166  (6.5) | 1084 (28.4) | 1509 (39.5) | 988  (25.9) | 235  (6.2) |
| Fatigue | 430  (34.2) | 216  (17.2) | 453  (36.0) | 159  (12.6) | 915  (35.8) | 460  (18.0) | 852  (33.3) | 331 (12.9) | 1345 (35.2) | 676  (17.7) | 1305 (34.2) | 490  (12.8) |
| Headache | 475  (37.8) | 283  (22.5) | 381  (30.3) | 119  (9.5) | 1006 (39.3) | 588  (23.0) | 695  (27.2) | 269  (10.5) | 1481 (38.8) | 871  (22.8) | 1076 (28.2) | 388  (10.2) |
| Body aches or joint pain | 569  (45.2) | 196  (15.6) | 356  (28.3) | 137  (10.9) | 1204 (47.1) | 381  (14.9) | 681  (26.6) | 292  (11.4) | 1773 (46.5) | 577  (15.1) | 1037 (27.2) | 429  (11.2) |
| Loss of taste/smell | 669  (53.2) | 87  (6.9) | 180  (14.3) | 322  (25.6) | 1435 (56.1) | 175  (6.8) | 413  (16.1) | 535  (20.9) | 2104 (55.1) | 262  (6.9) | 593  (15.5) | 857  (22.5) |
| Chills | 787  (62.6) | 220  (17.5) | 195  (15.5) | 56  (4.5) | 1660 (64.9) | 403  (15.8) | 387  (15.1) | 108  (4.2) | 2447 (64.1) | 623  (16.3) | 582  (15.3) | 164  (4.3) |
| Runny nose | 812  (64.5) | 257  (20.4) | 161  (12.8) | 28  (2.2) | 1571 (61.4) | 592  (23.1) | 347  (13.6) | 48  (1.9) | 2383 (62.4) | 849  (22.2) | 508  (13.3) | 76  (2.0) |
| Sore throat | 827  (65.7) | 256  (20.3) | 146  (11.6) | 29  (2.3) | 1692 (66.1) | 521  (20.4) | 290  (11.3) | 55  (2.2) | 2519 (66.0) | 777  (20.4) | 436  (11.4) | 84  (2.2) |
| Loss of appetite | 839  (66.7) | 113  (9.0) | 231  (18.4) | 75  (6.0) | 1707 (66.7) | 268  (10.5) | 449  (17.6) | 134  (5.2) | 2546 (66.7) | 381  (10.0) | 680  (17.8) | 209  (5.5) |
| Feeling feverish | 878  (69.8) | 209  (16.6) | 140  (11.1) | 31  (2.5) | 1783 (69.7) | 423  (16.5) | 299  (11.7) | 53  (2.1) | 2661 (69.7) | 632  (16.6) | 439  (11.5) | 84  (2.2) |
| Sneezing | 903  (71.8) | 234  (18.6) | 107  (8.5) | 14  (1.1) | 1838 (71.9) | 474 ( 18.5) | 228  (8.9) | 18  (0.7) | 2741 (71.8) | 708  (18.6) | 335  (8.8) | 32  (0.8) |
| Shortness of breath | 952  (75.7) | 164  (13.0) | 125  (9.9) | 17  (1.4) | 1957 (76.5) | 315  (12.3) | 258  (10.1) | 28  (1.1) | 2909 (76.2) | 479  (12.6) | 383  (10.0) | 45  (1.2) |
| Diarrhea | 976  (77.6) | 125  (9.9) | 140  (11.1) | 17  (1.4) | 2020 (79.0) | 275  (10.8) | 198  (7.7) | 65  (2.5) | 2996 (78.5) | 400  (10.5) | 338 (8.9) | 82  (2.1) |
| Sputum/phlegm | 980  (77.9) | 174  (13.8) | 88  (7.0) | 16  (1.3) | 1978 (77.3) | 325  (12.7) | 231  (9.0) | 24  (0.9) | 2958 (77.5) | 499  (13.1) | 319  (8.4) | 40  (1.0) |
| Nausea | 992  (78.9) | 145  (11.5) | 103  (8.2) | 18  (1.4) | 1990 (77.8) | 329  (12.9) | 208  (8.1) | 31  (1.2) | 2982 (78.1) | 474  (12.4) | 311  (8.1) | 49  (1.3) |
| Red or watery eyes | 1030 (81.9) | 134  (10.7) | 73  (5.8) | 21  (1.7) | 2048 (80.1) | 283  (11.1) | 197  (7.7) | 30  (1.2) | 3078 (80.7) | 417  (10.9) | 270  (7.1) | 51  (1.3) |
| Dizziness | 1039 (82.6) | 111  (8.8) | 95  (7.6) | 13  (1.0) | 2108 (82.4) | 255  (10.0) | 171  (6.7) | 24  (0.9) | 3147 (82.5) | 366  (9.6) | 266  (7.0) | 37  (1.0) |
| Pressure/ tightness in chest | 1059 (84.2) | 96  (7.6) | 90  (7.2) | 13  (1.0) | 2122 (83.0) | 241 (9.4) | 182  (7.1) | 13  (0.5) | 3181 (83.4) | 337  (8.8) | 272  (7.1) | 26  (0.7) |
| Stomachache | 1096 (87.1) | 82  (6.5) | 69  (5.5) | 11  (0.9) | 2246 (87.8) | 158  (6.2) | 127  (5.0) | 27  (1.1) | 3342 (87.6) | 240  (6.3) | 196  (5.1) | 38  (1.0) |
| Chest pain | 1166 (92.7) | 38  (3.0) | 46  (3.7) | 8  (0.6) | 2358 (92.2) | 98  (3.8) | 93  (3.6) | 9  (0.4) | 3524 (92.3) | 136  (3.6) | 139  (3.6) | 17  (0.4) |
| Confusion | 1169 (92.9) | 46  (3.7) | 41  (3.3) | 2  (0.2) | 2382 (93.1) | 111  (4.3) | 58  (2.3) | 7  (0.3) | 3551 (93.1) | 157  (4.1) | 99  (2.6) | 9  (0.2) |
| Vomiting | 1208 (96.0) | 26  (2.1) | 22  (1.7) | 2  (0.2) | 2469 (96.5) | 54 (2.1) | 23  (0.9) | 12  (0.5) | 3677 (96.4) | 80  (2.1) | 45  (1.2) | 14  (0.4) |
| Rash | 1238 (98.4) | 12  (1.0) | 6  (0.5) | 2  (0.2) | 2504 (97.9) | 30  (1.2) | 19  (0.7) | 5  (0.2) | 3742 (98.1) | 42  (1.1) | 25  (0.7) | 7  (0.2) |

**D** Immunoglobulin summary

| ***n* (%)** | **Placebo (*n* = 1258)** | **CAS + IMD (*n* = 2558)** | **Total (*N* = 3816)** |
| --- | --- | --- | --- |
| Baseline serology status |  |  |  |
| Positive | 364 (28.9) | 760 (29.7) | 1124 (29.5) |
| Negative | 860 (68.4) | 1729 (67.6) | 2589 (67.8) |
| Missing | 34 (2.7) | 69 (2.7) | 103 (2.7) |
| Baseline anti-S IgA status |  |  |  |
| Positive | 335 (26.6) | 672 (26.3) | 1007 (26.4) |
| Negative | 855 (68.0) | 1741 (68.1) | 2596 (68.0) |
| Missing | 68 (5.4) | 145 (5.7) | 213 (5.6) |
| Baseline anti-S IgG status |  |  |  |
| Positive | 135 (10.7) | 292 (11.4) | 427 (11.2) |
| Negative | 1055 (83.9) | 2120 (82.9) | 3175 (83.2) |
| Missing | 68 (5.4) | 146 (5.7) | 214 (5.6) |
| Baseline anti-N IgG status |  |  |  |
| Positive | 141 (11.2) | 312 (12.2) | 453 (11.9) |
| Negative | 1069 (85.0) | 2160 (84.4) | 3229 (84.6) |
| Missing | 3 (0.2) | 3 (0.1) | 6 (0.2) |
| Baseline NAb status |  |  |  |
| *n* | 364 | 760 | 1124 |
| Positive | 229 (62.9) | 492 (64.7) | 721 (64.1) |
| Negative | 123 (33.8) | 237 (31.2) | 360 (32.0) |
| Borderline | 4 (1.1) | 16 (2.1) | 20 (1.8) |
| Missing | 8 (2.2) | 15 (2.0) | 23 (2.0) |

Individuals were classified as seronegative if all available test results were negative, seropositive if ≥ 1 available test result was positive, missing if no test results were available, and borderline if there was ≥ 1 borderline test result (in the absence of any positive test result).

Anti-N, anti-nucleocapsid; Anti-S, anti-spike; Ig, immunoglobulin; NAb, neutralizing antibody.

Supplemental Table 3. Outcome of hospitalization and/or death in different subgroups

|  | Hospitalization and/or all-cause mortality | |
| --- | --- | --- |
| Subgroups, *n* (%) | Yes  *n* = 68 | No  *n* = 3748 |
| Obesity group |  |  |
| BMI ≥ 30 kg/m^2^ | 40 (1.8) | 2214 (98) |
| BMI ≥ 40 kg/m^2^ | 7 (1.9) | 371 (98) |
| Age group |  |  |
| ≥ 50 | 54 (2.8) | 1853 (97) |
| ≥ 65 | 20 (4.4) | 435 (96) |
| Race |  |  |
| White | 61 (1.9) | 3195 (98) |
| Black or African American | 2 (1.0) | 197 (99) |
| Asian, American Indian, or Alaska Native | 3 (1.6) | 180 (98) |
| Unknown or not reported | 2 (1.1) | 176 (99) |
| Ethnicity |  |  |
| Hispanic or Latino | 21 (1.5) | 1367 (98) |
| Not Hispanic or Latino | 46 (1.9) | 2354 (98) |
| Not reported | 1 (3.6) | 27 (96) |
| Sex |  |  |
| Female | 24 (1.2) | 1953 (99) |
| Male | 44 (2.4) | 1795 (98) |
| Viral load |  |  |
| ≤ 10^7^ copies/mL | 28 (1.5) | 1899 (99) |
| > 10^7^ copies/mL | 40 (2.1) | 1849 (98) |
| Risk factor: cardiovascular disease |  |  |
| No | 31 (1.3) | 2406 (99) |
| Yes | 37 (2.8) | 1307 (97) |
| Risk factor: chronic lung disease |  |  |
| No | 55 (1.7) | 3105 (98) |
| Yes | 13 (2.1) | 608 (98) |
| Risk factor: chronic metabolic disease |  |  |
| No | 49 (1.5) | 3169 (98) |
| Yes | 19 (3.4) | 544 (97) |
| Risk factor: immunosuppressed |  |  |
| No | 67 (1.8) | 3610 (98) |
| Yes | 1 (1.0) | 103 (99) |
| Risk factor: chronic kidney disease |  |  |
| No | 64 (1.7) | 3671 (98) |
| Yes | 4 (8.7) | 42 (91) |
| Risk factor: chronic liver disease |  |  |
| No | 66 (1.8) | 3679 (98) |
| Yes | 2 (5.6) | 34 (94) |
| Risk factor: taking immunosuppressants |  |  |
| No | 44 (2.4) | 1821 (98) |
| Yes | 1 (3.7) | 26 (96) |
| Number of risk factors |  |  |
| 1 | 11 (0.6) | 1829 (99) |
| 2 | 24 (2.1) | 1141 (98) |
| 3 | 22 (3.9) | 548 (96) |
| 4 or more | 11 (4.6) | 226 (95) |

Percent by row.

BMI, body mass index.

Supplemental Table 4. Patient seroconversion by day 29

| **Characteristic** | **Anti-N IgG by day 29** | | | |
| --- | --- | --- | --- | --- |
|  | **Positive** | **Negative** | **Missing** | **Total** |
| Serology baseline, *n (%)* |  |  |  |  |
| Positive | 892 (79.4) | 81 (7.2) | 151 (13.4) | 1124 (100) |
| Negative | 1929 (74.5) | 362 (14.0) | 298 (11.5) | 2589 (100) |
| Missing | 75 (72.8) | 8 (7.8) | 20 (19.4) | 103 (100) |
| **Total** | 2896 | 451 | 469 | 3816 |

Anti-N, anti-nucleocapsid; Ig, immunoglobulin.

Supplemental Table 5. Multivariate regression between anti-S IgG, anti-S IgA, and NAbs

| **NAb-positive and seropositive** | **MLM1: Log_2__NAb titer (all subjects)** | | |
| --- | --- | --- | --- |
| **Predictors** | **Estimates** | **CI** | ***P*** |
| Intercept | 8.09 | 7.93–8.26 | **< 0.001** |
| Log_10_ ELISA IgG ratio | 2.11 | 1.90–2.32 | **< 0.001** |
| Log_10_ ELISA IgA ratio | 0.47 | 0.18–0.77 | **0.002** |
| Observations | 614 | | |
| R^2^ / R^2^ adjusted | 0.439 / 0.437 | | |

MLM1: Log_10__IgG – Log_10__IgA ≤ 0; *P* value = 3.16e-14 ***

NAb titers, viral neutralizing World Health Organization titer (IU/mL)

Ig, immunoglobulin; MLM, multivariate linear modeling; NAb, neutralizing antibody.

Supplemental Table 6. Summary of log_2_ neutralizing antibody titers at baseline by risk factors for hospitalization, age, sex, race, ethnicity, and viral load subgroups in Phase III patients

| **Baseline subgroups** | | **BMI ≥ 30 kg/m^2^** | | | **BMI < 30 kg/m^2^** | | | **BMI ≥ 40 kg/m^2^** | | |
| --- | --- | --- | --- | --- | --- | --- | --- | --- | --- | --- |
| **Statistics** | | ***n* (%)** | | **Mean (Median) [Q1, Q3]** | ***n* (%)** | | **Mean (Median) [Q1, Q3]** | ***n* (%)** | | **Mean (Median) [Q1, Q3]** |
| Overall in Phase III (*n* = 3493) | | 2044 |  |  | 1446 |  |  | 338 |  |  |
| Sero+  (*n* = 992) (28.4%) | | 606 (29.6) |  | 6.27 (6.40) [3.46,7.84] | 386 (26.7) |  | 5.98 (5.98) [3.46,7.69] | 102 (30.2) |  | 6.24 (6.44) [3.46,8.03] |
| NAb | Positive (*n* = 635) (64%) |  | 402 (66.3) | 7.56 (7.33) [6.32,8.34] |  | 233 (60.4) | 7.46 (7.17) [6.17,8.26] |  | 70 (68.6) | 7.50 (7.32) [6.39,8.52] |
|  | Borderline (*n* = 9) (1%) |  | 7 (1.2) | 5.99 (5.99) [5.50,6.64] |  | 2 (0.5) | 7.87 (7.87) [7.65,8.09] |  | 1 (1) | 5.52 (5.52) [5.52,5.52] |
|  | Negative (*n* = 327) (33%) |  | 186 (30.7) | 3.52 (3.46) [3.46,3.46] |  | 141 (36.5) | 3.48 (3.46) [3.46,3.46] |  | 30 (29.4) | 3.46 (3.46) [3.46,3.46] |
| **Baseline subgroups** | | **BMI <40 kg/m^2^** | | | **Age ≥ 50 years** | | | **Age < 50 years** | | |
| **Statistics** | | ***n* (%)** | | **Mean (Median) [Q1, Q3]** | ***n* (%)** | | **Mean (Median) [Q1, Q3]** | ***n* (%)** | | **Mean (Median) [Q1, Q3]** |
| Overall in Phase III | | 3152 |  |  | 1752 |  |  | 1741 |  |  |
| Sero+ | | 890 (28.2) |  | 6.15 (6.19) [3.46,7.75] | 473 (27) |  | 6.13 (6.02) [3.46,7.92] | 519 (29.8) |  | 6.18 (6.39) [3.46,7.64] |
| NAb | Positive |  | 565 (63.5) | 7.53 (7.29) [6.21,8.28] |  | 290 (61.3) | 7.64 (7.55) [6.21,8.38] |  | 345 (66.5) | 7.42 (7.22) [6.30,8.20] |
|  | Borderline |  | 8 (0.9) | 6.59 (6.70) [5.98,7.87] |  | 5 (1.1) | 6.40 (6.92) [5.01,8.31] |  | 4 (0.8) | 6.52 (6.58) [6.22,6.88] |
|  | Negative |  | 297 (33.4) | 3.51 (3.46) [3.46,3.46] |  | 170 (35.9) | 3.53 (3.46) [3.46,3.46] |  | 157 (30.3) | 3.47 (3.46) [3.46,3.46] |
| **Baseline subgroups** | | **Age ≥ 65 years** | | | **Age < 65 years** | | | **Cardiovascular disease, including hypertension** | | |
| **Statistics** | | ***n* (%)** | | **Mean (Median) [Q1, Q3]** | ***n* (%)** | | **Mean (Median) [Q1, Q3]** | ***n* (%)** | | **Mean (Median) [Q1, Q3]** |
| Overall in Phase III | | 422 |  |  | 3071 |  |  | 1231 |  |  |
| Sero+ | | 129 (30.6) |  | 6.03 (5.51) [3.46,7.71] | 863 (28.1) |  | 6.18 (6.27) [3.46,7.79] | 329 (26.7) |  | 6.24 (6.27) [3.46,7.95] |
| NAb | Positive |  | 72 (55.8) | 7.77 (7.48) [6.43,8.58] |  | 563 (65) | 7.49 (7.27) [6.25,8.29] |  | 211 (64.1) | 7.69 (7.55) [6.36,8.64] |
|  | Borderline |  | 2 (1.6) | 8.31 (8.31) [8.31,8.31] |  | 7 (0.8) | 6.20 (6.46) [5.51,7.06] |  | 4 (1.2) | 5.47 (5.52) [4.49,6.47] |
|  | Negative |  | 51 (39.5) | 3.53 (3.46) [3.46,3.46] |  | 276 (32) | 3.50 (3.46) [3.46,3.46] |  | 110 (33.4) | 3.50 (3.46) [3.46,3.46] |
| **Baseline subgroups** | | **Chronic lung disease, including asthma** | | | **Chronic metabolic disease, including diabetes** | | | **Immunocompromised** | | |
| **Statistics** | | ***n* (%)** | | **Mean (Median) [Q1, Q3]** | ***n* (%)** | | **Mean (Median) [Q1, Q3]** | ***n* (%)** | | **Mean (Median) [Q1, Q3]** |
| Overall in Phase III | | 573 |  |  | 490 |  |  | 95 |  |  |
| Sero+ | | 161 (28.1) |  | 6.31 (6.21) [3.46,7.98] | 168 (34.3) |  | 6.58 (6.85) [3.46,7.98] | 23 (24.2) |  | 5.61 (4.85) [3.46,7.04] |
| NAb | Positive |  | 107 (66.5) | 7.59 (7.26) [6.11,8.56] |  | 118 (70.2) | 7.73 (7.52) [6.49,8.66] |  | 12 (52.2) | 7.40 (6.97) [5.36,8.34] |
|  | Borderline |  | 1 (0.6) | NA |  | 2 (1.2) | 5.99 (5.99) [5.76,6.23] |  | 0 | NA |
|  | Negative |  | 49 (30.4) | 3.46 (3.46) [3.46,3.46] |  | 43 (25.6) | 3.51 (3.46) [3.46,3.46] |  | 10 (43.5) | 3.46 (3.46) [3.46,3.46] |
| **Baseline subgroups** | | **Chronic kidney disease, including those on dialysis** | | | **Chronic liver disease** | | | **Taking immunosuppressants** | | |
| **Statistics** | | ***n* (%)** | | **Mean (Median) [Q1, Q3]** | ***n* (%)** | | **Mean (Median) [Q1, Q3]** | ***n* (%)** | | **Mean (Median) [Q1, Q3]** |
| Overall in Phase III | | 37 |  |  | 33 |  |  | 21 |  |  |
| Sero+ | | 9 (24.3) |  | 4.78 (3.46) [3.46,6.36] | 9 (27.3) |  | 6.88 (6.36) [6.02,7.82] | 6 (28.6) |  | 5.01 (4.31) [3.46,6.11] |
| NAb | Positive |  | 4 (44.4) | 6.43 (6.57) [6.06,6.94] |  | 6 (66.7) | 8.15 (7.28) [6.45,9.03] |  | 3 (50) | 6.56 (6.43) [5.80,7.25] |
|  | Borderline |  | 0 | NA |  | 0 | NA |  | 0 | NA |
|  | Negative |  | 5 (55.6) | 3.46 (3.46) [3.46,3.46] |  | 2  (22.2) | 3.46 (3.46) [3.46,3.46] |  | 3 (50) | 3.46 (3.46) [3.46,3.46] |
| **Baseline subgroups** | | **Race – White** | | | **Race – Black or African American** | | | **Race – Asian, American Indian, or Alaska Native** | | |
| **Statistics** | | ***n* (%)** | | **Mean (Median) [Q1, Q3]** | ***n* (%)** | | **Mean (Median) [Q1, Q3]** | ***n* (%)** | | **Mean (Median) [Q1, Q3]** |
| Overall in Phase III | | 2983 |  |  | 168 |  |  | 176 |  |  |
| Sero+ | | 843 (28.3%) |  | 6.12 (6.10) [3.46,7.75] | 56 (33.3) |  | 6.44 (7.10) [3.46,8.01] | 48 (27.3) |  | 5.94 (6.02) [3.46,7.82] |
| NAb | Positive |  | 533 (63.2) | 7.52 (7.26) [6.21,8.34] |  | 38 (67.9) | 7.68 (7.69) [6.82,8.19] |  | 27 (56.2) | 7.47 (7.08) [6.19,8.03] |
|  | Borderline |  | 8 (0.9) | 6.20 (6.46) [5.51,7.06] |  | 1 (1.8) | 8.32 (8.32) [8.32,8.32] |  | 0 | NA |
|  | Negative |  | 286 (33.9) | 3.51 (3.46) [3.46,3.46] |  | 16 (28.6) | 3.46 (3.46) [3.46,3.46] |  | 17 (35.4) | 3.46 (3.46) [3.46,3.46] |
| **Baseline subgroups** | | **Race – Unknown or not reported** | | | **Sex – Female** | | | **Sex – Male** | | |
| **Statistics** | | ***n* (%)** | | **Mean (Median) [Q1, Q3]** | ***n* (%)** | | **Mean (Median) [Q1, Q3]** | ***n* (%)** | | **Mean (Median) [Q1, Q3]** |
| Overall in Phase III | | 166 |  |  | 1804 |  |  | 1689 |  |  |
| Sero+ | | 45 (27.1) |  | 6.70 (6.89) [5.49,7.77] | 520 (28.8) |  | 6.42 (6.55) [3.46,7.90] | 472 (27.9) |  | 5.87 (5.71) [3.46,7.47] |
| NAb | Positive |  | 37 (82.2) | 7.40 (7.16) [6.54,7.88] |  | 363 (69.8) | 7.57 (7.40) [6.34,8.30] |  | 272 (57.6) | 7.46 (7.17) [6.17,8.31] |
|  | Borderline |  | 0 | NA |  | 3 (0.6) | 6.15 (5.52) [5.51,6.47] |  | 6 (1.3) | 6.65 (6.70) [6.46,8.31] |
|  | Negative |  | 8 (17.8) | 3.46 (3.46) [3.46,3.46] |  | 143 (27.5) | 3.50 (3.46) [3.46,3.46] |  | 184 (39) | 3.51 (3.46) [3.46,3.46] |
| **Baseline subgroups** | | **Ethnic – Hispanic or Latino** | | | **Ethnic – Not Hispanic or Latino** | | | **Ethnic – Not reported** | | |
| **Statistics** | | ***n* (%)** | | **Mean (Median) [Q1, Q3]** | ***n* (%)** | | **Mean (Median) [Q1, Q3]** | ***n* (%)** | | **Mean (Median) [Q1, Q3]** |
| Overall in Phase III | | 1238 |  |  | 2227 |  |  | 28 |  |  |
| Sero+ | | 472 (38.1) |  | 6.55 (6.74) [3.46,7.99] | 513 (23) |  | 5.79 (5.55) [3.46,7.48] | 7 (25) |  | 6.50 (6.78) [4.19,8.17] |
| NAb | Positive |  | 345 (73.1) | 7.56 (7.32) [6.27,8.34] |  | 285 (55.6) | 7.47 (7.25) [6.23,8.20] |  | 5 (71.4) | 8.02 (7.84) [6.98,8.88] |
|  | Borderline |  | 4 (0.8) | 7.23 (7.06) [6.64,7.65] |  | 5 (1) | 5.70 (5.51) [4.98,6.22] |  | 0 | NA |
|  | Negative |  | 116 (24.6) | 3.52 (3.46) [3.46,3.46] |  | 209 (40.7) | 3.50 (3.46) [3.46,3.46] |  | 2 (28.6) | 3.46 (3.46) [3.46,3.46] |
| **Baseline subgroups** | | **< =10^7 copies/mL** | | | **> 10^7 copies/mL** | | |  |  |  |
| **Statistics** | | ***n* (%)** | | **Mean (Median) [Q1, Q3]** | ***n* (%)** | | **Mean (Median) [Q1, Q3]** |  |  |  |
| Overall in Phase III | | 1757 |  |  | 1736 |  |  |  |  |  |
| Sero+ | | 744 (42.3) |  | 6.71 (6.86) [5.13,8.04] | 218 (12.6) |  | 4.20 (3.46) [3.46,3.46] |  |  |  |
| NAb | Positive |  | 589 (79.2) | 7.58 (7.34) [6.36,8.33] |  | 46 (21.1) | 6.80 (6.09) [5.19,7.82] |  |  |  |
|  | Borderline |  | 7 (0.9) | 7.12 (7.06) [6.52,8.09] |  | 2 (0.9) | 4.48 (4.48) [3.97,4.98] |  |  |  |
|  | Negative |  | 162 (21.8) | 3.54 (3.46) [3.46,3.46] |  | 165 (75.7) | 3.47 (3.46) [3.46,3.46] |  |  |  |
| **Baseline subgroups** | | **No. of risk factor = 1** | | | **No. of risk factor = 2** | | | **No. of risk factor = 3** | | |
| **Statistics** | | ***n* (%)** | | **Mean (Median) [Q1, Q3]** | ***n* (%)** | | **Mean (Median) [Q1, Q3]** | ***n* (%)** | | **Mean (Median) [Q1, Q3]** |
| Overall in Phase III | | 1686 |  |  | 1087 |  |  | 516 |  |  |
| Sero+ | | 478 (28.4) |  | 6.11 (6.21) [3.46,7.55] | 301 (27.7) |  | 6.00 (6.04) [3.46,7.77] | 160 (31) |  | 6.31 (6.41) [3.46,8.09] |
| NAb | Positive |  | 306 (64) | 7.43 (7.15) [6.20,8.20] |  | 185 (61.5) | 7.44 (7.32) [6.23,8.19] |  | 107 (66.9) | 7.67 (7.70) [6.39,8.67] |
|  | Borderline |  | 4 (0.8) | 6.98 (7.06) [6.40,7.65] |  | 2 (0.7) | 7.39 (7.39) [6.92,7.85] |  | 1 (0.6) | 3.46 (3.46) [3.46,3.46] |
|  | Negative |  | 155 (32.4) | 3.48 (3.46) [3.46,3.46] |  | 109 (36.2) | 3.54 (3.46) [3.46,3.46] |  | 50 (31.2) | 3.46 (3.46) [3.46,3.46] |
| **Baseline subgroups** | | **No. of risk factor = 4 or more** | | |  |  |  |  |  |  |
| **Statistics** | | n (%) | | Mean (Median) [Q1, Q3] |  |  |  |  |  |  |
| Overall in Phase III | | 200 |  |  |  |  |  |  |  |  |
| Sero+ |  | 53 (26.5) |  | 7.04 (6.99) [4.21,9.28] |  |  |  |  |  |  |
| NAb | Positive |  | 37 (69.8) | 8.28 (7.83) [6.74,10.17] |  |  |  |  |  |  |
|  | Borderline |  | 2 (3.8) | 5.52 (5.52) [5.52,5.52] |  |  |  |  |  |  |
|  | Negative |  | 13 (24.5) | 3.63 (3.46) [3.46,3.46] |  |  |  |  |  |  |

Missingness of NAb titers and NAb qualitative is not reported in this table.
NAb titers, viral neutralizing World Health Organization titer (IU/mL).
BMI, body mass index; NAb, neutralizing antibody; Sero+, seropositive.

Supplemental Table 7. Summary of number of risk factors and specific age groups at baseline by race and ethnic group

**A** By race

| **Variables** |  | **Overall (*N* = 3816)** | **White (*n* = 3256)** | **Black or African American (*n* = 199)** | **Asian, American Indian, or Alaska Native (*n* = 183)** | **Unknown or not reported (*n* = 178)** |
| --- | --- | --- | --- | --- | --- | --- |
| No. of risk factors | Mean (median) | 1.81 (2.00) | 1.81 (2.00) | 1.93 (2.00) | 1.74 (1.00) | 1.68 (1.00) |
| 1 | *n* (%) | 1840 (48) | 1568 (48) | 82 (41) | 93 (51) | 97 (54) |
| 2 | *n* (%) | 1165 (31) | 994 (31) | 67 (34) | 55 (30) | 49 (28) |
| 3 | *n* (%) | 570 (15) | 485 (15) | 34 (17) | 25 (14) | 26 (15) |
| 4 | *n* (%) | 237 (6.2) | 206 (6.3) | 16 (8.0) | 9 (4.9) | 6 (3.4) |
| Age group |  |  |  |  |  |  |
| ≥ 50 | *n* (%) | 1907 (50) | 1661 (51) | 73 (37) | 94 (51) | 79 (44) |
| ≥ 65 | *n* (%) | 455 (12) | 396 (12) | 6 (3.0) | 33 (18) | 20 (11) |

**B** By ethnicity

| **Variables** |  | **Overall (*N* = 3816)** | **Hispanic or Latino (*n* = 1388)** | **Not Hispanic or Latino (*n* = 2400)** | **Not reported (*n* = 28)** |
| --- | --- | --- | --- | --- | --- |
| No. of risk factors | Mean (median) | 1.81 (2.00) | 1.73 (1.00) | 1.86 (2.00) | 1.57 (1.00) |
| 1 | *n* (%) | 1840 (48) | 714 (51) | 1111 (46) | 15 (54) |
| 2 | *n* (%) | 1165 (31) | 419 (30) | 736 (31) | 10 (36) |
| 3 | *n* (%) | 570 (15) | 184 (13) | 383 (16) | 3 (11) |
| 4 | *n* (%) | 237 (6.2) | 68 (4.9) | 169 (7.0) | 0 (0) |
| Age group |  |  |  |  |  |
| ≥ 50 | *n* (%) | 1907 (50) | 581 (42) | 1,309 (55) | 17 (61) |
| ≥ 65 | *n* (%) | 455 (12) | 109 (7.9) | 345 (14) | 1 (3.6) |

Note that the catalog of risk factors is followed by Table S1

Supplemental Table 8. Treatment effects (probability difference) and associated 95% confidence band obtained using a 2-step approach for symptoms

| **Day** | **Cough** | **Fatigue** | **Body aches or joint pain** | **Loss of appetite** | **Chills** |
| --- | --- | --- | --- | --- | --- |
| 1 | –2.3 (–6.0, 1.5) | –2.1 (–5.9, 1.7) | –2.1 (–7.0, 2.8) | –0.5 (–5.3, 4.3) | –2.5 (–6.4, 1.5) |
| 2 | –3.7 (–7.5, 0.1) | –3.4 (–6.9, 0.2) | –3.4 (–7.7, 0.9) | –3.1 (–6.9, 0.7) | –3.5 (–7.1, 0.1) |
| 3 | –4.0 (–7.9, –0.1)* | –5.4 (–9.0, –1.8)* | –8.1 (–12.9, –3.2)* | –4.8 (–8.3, –1.3)* | –8.4 (–12.3, –4.5)* |
| 4 | –4.6 (–8.3, –0.8)* | –7.0 (–10.7, –3.3)* | –11.1 (–15.7, –6.5)* | –6.8 (–10.9, –2.8)* | –9.3 (–12.0, –6.6)* |
| 5 | –6.8 (–10.9, –2.8)* | –7.5 (–11.4, –3.6)* | –9.2 (–13.2, –5.2)* | –6.6 (–10.3, –2.9)* | –8.9 (–11.6, –6.1)* |
| 6 | –7.5 (–11.4, –3.6)* | –7.8 (–11.7, –3.8)* | –9.6 (–13.7, –5.5)* | –6.1 (–9.4, –2.9)* | –7.8 (–10.4, –5.3)* |
| 7 | –8.2 (–12.1, –4.2)* | –8.8 (–12.9, –4.7)* | –8.3 (–12.4, –4.2)* | –6.9 (–10.1, –3.7)* | –7.2 (–9.6, –4.7)* |
| 8 | –8.7 (–12.6, –4.7)* | –9.5 (–13.8, –5.2)* | –6.8 (–10.6, –3.0)* | –7.3 (–10.3, –4.4)* | –6.4 (–8.6, –4.2)* |
| 9 | –9.5 (–13.5, –5.5)* | –9.1 (–13.4, –4.8)* | –5.3 (–8.9, –1.7)* | –7.2 (–9.9, –4.6)* | –5.3 (–7.3, –3.3)* |
| 10 | –10.2 (–14.3, –6.0)* | –8.6 (–12.9, –4.2)* | –4.1 (–7.5, –0.7)* | –6.1 (–8.9, –3.3)* | –4.3 (–6.1, –2.4)* |
| 11 | –10.3 (–14.5, –6.1)* | –7.8 (–11.9, –3.7)* | –3.3 (–6.5, 0.0) | –5.4 (–8.0, –2.7)* | –3.2 (–5.0, –1.5)* |
| 12 | –10.9 (–15.1, –6.7)* | –6.6 (–10.6, –2.7)* | –2.9 (–6.1, 0.3) | –5.4 (–7.9, –2.8)* | –2.3 (–3.8, –0.8)* |
| 13 | –10.8 (–15.0, –6.6)* | –6.1 (–10.1, –2.1)* | –1.4 (–4.1, 1.4) | –4.4 (–6.6, –2.1)* | –2.1 (–3.6, –0.7)* |
| 14 | –9.6 (–13.7, –5.5)* | –5.7 (–9.5, –1.8)* | –2.0 (–5.0, 1.0) | –3.5 (–5.7, –1.4)* | –1.6 (–3.0, –0.1)* |
| 15 | –8.3 (–12.4, –4.1)* | –4.9 (–8.7, –1.2)* | –1.6 (–4.5, 1.4) | –3.3 (–5.4, –1.3)* | –1.5 (–3.0, 0.0) |

| **Day** | **Headache** | **Feeling feverish** | **Loss of taste/smell** | **Shortness of breath** | **Diarrhea** |
| --- | --- | --- | --- | --- | --- |
| 1 | –1.4 (–5.8, 2.9) | 0.4 (–3.2, 4.0) | –2.0 (–6.3, 2.3) | –0.8 (–4.2, 2.5) | –1.5 (–4.6, 1.6) |
| 2 | –3.1 (–7.1, 1.0) | –1.1 (–4.5, 2.2) | –2.2 (–6.3, 2.0) | –1.5 (–4.8, 1.9) | –2.1 (–5.2, 1.0) |
| 3 | –6.7 (–11.4, –1.9)* | –7.8 (–11.4, –4.3)* | –2.4 (–6.5, 1.7) | –2.3 (–5.7, 1.0) | –3.4 (–6.4, –0.3)* |
| 4 | –7.0 (–10.8, –3.2)* | –10.0 (–12.8, –7.1)* | –3.6 (–7.8, 0.6) | –3.0 (–6.3, 0.4) | –4.4 (–7.4, –1.4)* |
| 5 | –8.2 (–13.0, –3.5)* | –9.6 (–12.3, –6.8)* | –4.9 (–9.1, –0.8)* | –4.4 (–7.7, –1.2)* | –4.9 (–7.7, –2.2)* |
| 6 | –5.5 (–9.2, –1.8)* | –8.4 (–11.0, –5.7)* | –5.9 (–10.1, –1.8)* | –4.8 (–8.0, –1.5)* | –5.2 (–8.0, –2.4)* |
| 7 | –6.2 (–10.1, –2.4)* | –7.0 (–9.5, –4.5)* | –6.2 (–10.3, –2.0)* | –5.2 (–8.5, –1.9)* | –4.7 (–7.2, –2.1)* |
| 8 | –6.0 (–9.7, –2.2)* | –6.1 (–8.3, –3.9)* | –5.7 (–9.8, –1.5)* | –5.5 (–8.8, –2.1)* | –3.6 (–6.1, –1.2)* |
| 9 | –4.5 (–8.3, –0.7)* | –5.2 (–7.1, –3.3)* | –5.2 (–9.1, –1.3)* | –5.1 (–8.2, –2.0)* | –2.7 (–5.0, –0.5)* |
| 10 | –3.5 (–7.2, 0.2) | –4.3 (–6.0, –2.6)* | –5.4 (–9.2, –1.6)* | –5.5 (–8.5, –2.5)* | –3.2 (–5.3, –1.0)* |
| 11 | –3.1 (–6.6, 0.4) | –3.2 (–4.7, –1.6)* | –6.0 (–9.7, –2.2)* | –5.5 (–8.4, –2.6)* | –3.2 (–5.2, –1.1)* |
| 12 | –3.4 (–7.0, 0.1) | –2.2 (–3.6, –0.8)* | –6.2 (–10.0, –2.3)* | –4.4 (–7.3, –1.6)* | –2.6 (–4.6, –0.7)* |
| 13 | –4.0 (–7.3, –0.6)* | –1.5 (–2.8, –0.2)* | –5.4 (–9.1, –1.7)* | –3.8 (–6.6, –1.0)* | –2.5 (–4.4, –0.6)* |
| 14 | –4.5 (–7.7, –1.2)* | –1.1 (–2.3, 0.2) | –4.9 (–8.6, –1.2)* | –3.7 (–6.4, –0.9)* | –2.3 (–4.1, –0.5)* |
| 15 | –4.8 (–8.1, –1.5)* | –1.0 (–2.3, 0.3) | –4.4 (–8.1, –0.7)* | –4.1 (–7.1, –1.2)* | –2.1 (–3.9, –0.3)* |

| **Day** | **Nausea** | **Sputum/phlegm** | **Pressure/tightness in chest** | **Sore throat** | **Dizziness** |
| --- | --- | --- | --- | --- | --- |
| 1 | 0.6 (–3.2, 4.5) | 0.3 (–3.3, 3.8) | 1.3 (–2.4, 5.0) | 0.0 (–4.0, 4.0) | 0.1 (–2.8, 3.0) |
| 2 | –1.3 (–4.4, 1.8) | –0.3 (–3.4, 2.9) | –1.2 (–4.2, 1.9) | 0.4 (–3.0, 3.7) | –0.2 (–2.8, 2.3) |
| 3 | –3.0 (–6.3, 0.2) | –1.3 (–4.4, 1.8) | –1.9 (–4.7, 0.8) | –0.5 (–3.6, 2.5) | –1.2 (–3.8, 1.4) |
| 4 | –3.5 (–6.7, –0.4)* | –1.8 (–4.9, 1.3) | –3.4 (–6.2, –0.7)* | –1.0 (–4.5, 2.4) | –1.9 (–4.5, 0.7) |
| 5 | –3.5 (–6.2, –0.8)* | –1.6 (–4.8, 1.7) | –3.7 (–6.4, –0.9)* | –2.9 (–6.0, 0.2) | –2.1 (–4.5, 0.3) |
| 6 | –4.6 (–7.7, –1.5)* | –1.8 (–5.1, 1.4) | –3.5 (–6.2, –0.8)* | –3.8 (–6.8, –0.7)* | –2.3 (–4.5, –0.1)* |
| 7 | –4.5 (–7.2, –1.8)* | –2.1 (–5.4, 1.2) | –2.7 (–5.3, –0.1)* | –4.3 (–7.3, –1.3)* | –2.8 (–4.9, –0.7)* |
| 8 | –4.5 (–7.0, –1.9)* | –2.8 (–6.2, 0.7) | –3.3 (–5.8, –0.9)* | –3.5 (–6.3, –0.7)* | –2.6 (–4.6, –0.6)* |
| 9 | –4.2 (–6.6, –1.8)* | –3.9 (–7.1, –0.6)* | –1.3 (–3.7, 1.2) | –2.5 (–5.0, 0.0) | –2.0 (–3.9, –0.1)* |
| 10 | –3.2 (–5.6, –0.8)* | –4.2 (–7.5, –0.8)* | –2.6 (–4.8, –0.3)* | –2.4 (–4.7, –0.1)* | –1.7 (–3.5, 0.0) |
| 11 | –2.5 (–4.7, –0.4)* | –4.2 (–7.4, –1.0)* | –2.6 (–4.8, –0.3)* | –2.5 (–4.9, –0.2)* | –1.9 (–3.7, –0.2)* |
| 12 | –2.0 (–4.0, 0.0) | –4.0 (–7.0, –1.0)* | –2.0 (–4.2, 0.1) | –2.2 (–4.5, 0.2) | –1.8 (–3.5, –0.2)* |
| 13 | –1.9 (–3.7, 0.0) | –3.6 (–6.4, –0.8)* | –2.2 (–4.4, –0.1)* | –2.0 (–4.3, 0.3) | –1.6 (–3.3, 0.0) |
| 14 | –1.9 (–3.6, –0.2)* | –2.8 (–5.6, 0.0) | –2.0 (–4.1, 0.1) | –1.9 (–4.1, 0.3) | –1.5 (–3.1, 0.2) |
| 15 | –1.3 (–3.0, 0.3) | –2.6 (–5.4, 0.3) | –1.9 (–4.0, 0.1) | –2.3 (–4.6, 0.1) | –1.5 (–3.2, 0.1) |

| **Day** | **Chest pain** | **Vomiting** | **Sneezing** | **Stomachache** | **Runny nose** |
| --- | --- | --- | --- | --- | --- |
| 1 | –0.1 (–2.1, 1.9) | –0.3 (–1.6, 1.0) | 0.5 (–3.1, 4.0) | –0.1 (–2.8, 2.7) | 4.3 (0.5, 8.0) |
| 2 | –0.5 (–2.2, 1.2) | –0.6 (–1.8, 0.6) | 0.4 (–2.7, 3.6) | –0.2 (–2.6, 2.2) | 4.2 (1.0, 7.5) |
| 3 | –0.9 (–2.6, 0.8) | –1.3 (–2.5, –0.1)* | –0.7 (–3.9, 2.6) | –0.7 (–2.8, 1.5) | 2.5 (–1.0, 6.0) |
| 4 | –1.1 (–2.8, 0.5) | –1.5 (–2.6, –0.4)* | –1.7 (–4.7, 1.2) | –1.4 (–3.4, 0.7) | 0.0 (–3.4, 3.3) |
| 5 | –1.1 (–2.6, 0.4) | –1.2 (–2.2, –0.3)* | –2.2 (–4.9, 0.5) | –1.8 (–3.8, 0.2) | –1.7 (–5.0, 1.6) |
| 6 | –1.0 (–2.5, 0.4) | –1.3 (–2.3, –0.3)* | –1.7 (–4.2, 0.8) | –2.0 (–4.0, 0.0) | –2.2 (–5.5, 1.0) |
| 7 | –1.2 (–2.7, 0.3) | –1.4 (–2.3, –0.4)* | –1.0 (–3.5, 1.5) | –1.7 (–3.4, 0.0) | –2.0 (–5.1, 1.1) |
| 8 | –1.6 (–3.1, 0.0) | –1.3 (–2.2, –0.3)* | –0.9 (–3.3, 1.5) | –1.1 (–2.6, 0.4) | –1.6 (–4.8, 1.5) |
| 9 | –1.8 (–3.3, –0.4)* | –1.0 (–1.8, –0.2)* | –0.9 (–3.3, 1.4) | –0.9 (–2.3, 0.5) | –1.2 (–4.1, 1.7) |
| 10 | –1.7 (–3.1, –0.3)* | –0.7 (–1.4, 0.0) | –1.0 (–3.3, 1.4) | –1.0 (–2.3, 0.4) | –0.7 (–3.6, 2.1) |
| 11 | –1.3 (–2.5, –0.1)* | –0.5 (–1.2, 0.1) | –1.1 (–3.4, 1.2) | –1.1 (–2.4, 0.2) | –0.7 (–3.5, 2.1) |
| 12 | –0.8 (–2.0, 0.3) | –0.4 (–1.0, 0.2) | –1.3 (–3.6, 1.0) | –1.0 (–2.2, 0.3) | –0.9 (–3.5, 1.8) |
| 13 | –0.5 (–1.7, 0.7) | –0.3 (–0.8, 0.2) | –0.8 (–2.9, 1.4) | –0.6 (–1.9, 0.7) | –1.2 (–3.9, 1.5) |
| 14 | –0.5 (–1.7, 0.7) | –0.3 (–0.8, 0.2) | –0.1 (–2.2, 1.9) | –0.4 (–1.7, 0.9) | –1.3 (–4.0, 1.5) |
| 15 | –0.6 (–1.8, 0.6) | –0.4 (–1.1, 0.2) | 0.1 (–1.9, 2.2) | –0.4 (–1.8, 1.0) | –1.2 (–3.9, 1.5) |

| **Day** | **Confusion** | **Rash** | **Red or watery eyes** |
| --- | --- | --- | --- |
| 1 | –0.3 (–2.2, 1.6) | 0.5 (–0.7, 1.8) | 2.1 (–1.1, 5.2) |
| 2 | –0.2 (–1.8, 1.3) | 0.7 (–0.5, 1.9) | 1.5 (–1.4, 4.3) |
| 3 | 0.2 (–1.4, 1.8) | 0.8 (–0.3, 1.8) | 0.1 (–2.9, 3.1) |
| 4 | 0.1 (–1.4, 1.5) | 0.3 (–0.6, 1.2) | –0.6 (–3.2, 2.0) |
| 5 | –0.2 (–1.5, 1.1) | –0.2 (–1.2, 0.8) | –0.5 (–3.0, 2.0) |
| 6 | –0.3 (–1.6, 0.9) | 0.1 (–0.9, 1.1) | –0.1 (–2.3, 2.1) |
| 7 | –0.6 (–1.9, 0.6) | –0.2 (–1.2, 0.8) | 0.4 (–1.8, 2.5) |
| 8 | –0.6 (–1.8, 0.6) | –0.2 (–1.2, 0.7) | 0.3 (–1.8, 2.3) |
| 9 | –0.3 (–1.4, 0.7) | –0.1 (–1.0, 0.8) | –0.1 (–2.1, 2.0) |
| 10 | –0.5 (–1.6, 0.5) | –0.1 (–1.0, 0.7) | –0.1 (–2.1, 1.8) |
| 11 | –0.7 (–1.8, 0.4) | –0.2 (–1.0, 0.6) | 0.0 (–1.8, 1.8) |
| 12 | –0.4 (–1.5, 0.7) | 0.0 (–0.7, 0.8) | 0.3 (–1.4, 2.0) |
| 13 | –0.3 (–1.3, 0.6) | 0.0 (–0.6, 0.7) | 0.3 (–1.3, 2.0) |
| 14 | –0.5 (–1.5, 0.5) | 0.1 (–0.5, 0.7) | 0.1 (–1.5, 1.8) |
| 15 | –0.5 (–1.7, 0.6) | –0.2 (–1.1, 0.6) | 0.0 (–1.7, 1.6) |

*Days achieving statistical significance.

Supplemental Table 9. Cox regression analysis of the relationship between severity of composite symptom variables and hospitalization/death

| **Symptom 1** | **Symptom 2** | **Baseline (per 1-point increase, e.g., from mild to moderate for cough and feeling feverish)** | | **Change from baseline (per 1-point decrease, e.g., from moderate to mild for cough and feeling feverish)** | |
| --- | --- | --- | --- | --- | --- |
|  |  | **HR (95% CI)** | ***P*-value** | **HR (95% CI)** | ***P*-value** |
| Cough | Feeling feverish | 5.54 (3.70–8.30) | < 0.001 | 0.22 (0.16–0.31) | < 0.001 |
| Shortness of breath | Feeling feverish | 4.93 (3.51–6.93) | < 0.001 | 0.25 (0.19–0.34) | < 0.001 |
| Shortness of breath | Cough | 4.79 (3.36–6.84) | < 0.001 | 0.25 (0.18–0.33) | < 0.001 |
| Cough | Fatigue | 4.38 (3.00–6.40) | < 0.001 | 0.26 (0.18–0.37) | < 0.001 |
| Cough | Loss of appetite | 4.33 (2.95–6.36) | < 0.001 | 0.27 (0.19–0.37) | < 0.001 |
| Shortness of breath | Loss of appetite | 4.13 (2.83–6.02) | < 0.001 | 0.31 (0.22–0.43) | < 0.001 |
| Feeling feverish | Fatigue | 4.07 (2.75–6.04) | < 0.001 | 0.32 (0.23–0.44) | < 0.001 |
| Shortness of breath | Fatigue | 3.84 (2.68–5.51) | < 0.001 | 0.32 (0.23–0.46) | < 0.001 |
| Feeling feverish | Loss of appetite | 3.95 (2.65–5.88) | < 0.001 | 0.34 (0.25–0.46) | < 0.001 |
| Fatigue | Loss of appetite | 3.16 (2.19–4.57) | < 0.001 | 0.41 (0.31–0.55) | < 0.001 |

For each pair of the 5 symptoms, a composite symptom variable was derived as taking the average of the 2 symptoms. A time-varying Cox regression model was fitted to each composite symptom variable individually, with time to hospitalization/death as outcome with the following covariates: baseline symptom score, daily symptom change from baseline, age, sex, BMI, treatment indicator, and baseline viral load (in log_10_). Reported *P*-values were adjusted using the Bonferroni approach for baseline and change from baseline, respectively.

Supplemental Table 10. COVID-19 symptoms assessed in this study

| **Number** | **Category** |
| --- | --- |
| 1 | Altered or loss of taste/smell |
| 2 | Fatigue |
| 3 | Cough |
| 4 | Sputum/phlegm |
| 5 | Loss of appetite |
| 6 | Runny nose |
| 7 | Chills |
| 8 | Diarrhea |
| 9 | Sneezing |
| 10 | Body aches such as muscle pain or joint pain |
| 11 | Feeling feverish |
| 12 | Pressure/tightness in chest |
| 13 | Dizziness |
| 14 | Nausea |
| 15 | Confusion |
| 16 | Stomachache |
| 17 | Vomiting |
| 18 | Rash |
| 19 | Sore throat |
| 20 | Red or watery eyes |
| 21 | Headache |
| 22 | Chest pain |
| 23 | Shortness of breath/difficulty breathing |

Supplemental Table 11. Proportion of patients with symptoms (i.e., mild/moderate/severe) from day 1 to day 29 by treatment arm

| **Day** | **Cough** | | **Fatigue** | | **Headache** | |
| --- | --- | --- | --- | --- | --- | --- |
|  | **Placebo** | **CAS + IMD** | **Placebo** | **CAS + IMD** | **Placebo** | **CAS + IMD** |
| 1 | 911/1258 (72.4) | 1821/2558 (71.2) | 828/1258 (65.8) | 1643/2558 (64.2) | 783/1258 (62.2) | 1552/2558 (60.7) |
| 2 | 771/1161 (66.4) | 1472/2353 (62.6) | 709/1161 (61.1) | 1368/2353 (58.1) | 623/1161 (53.7) | 1188/2353 (50.5) |
| 3 | 706/1151 (61.3) | 1386/2363 (58.7) | 671/1151 (58.3) | 1261/2363 (53.4) | 542/1151 (47.1) | 931/2363 (39.4) |
| 4 | 644/1123 (57.3) | 1262/2320 (54.4) | 628/1123 (55.9) | 1133/2320 (48.8) | 461/1123 (41.1) | 800/2320 (34.5) |
| 5 | 636/1119 (56.8) | 1158/2316 (50.0) | 596/1119 (53.3) | 1066/2316 (46.0) | 452/1119 (40.4) | 727/2316 (31.4) |
| 6 | 571/1094 (52.2) | 1046/2252 (46.4) | 532/1094 (48.6) | 982/2252 (43.6) | 365/1094 (33.4) | 671/2252 (29.8) |
| 7 | 523/1035 (50.5) | 953/2170 (43.9) | 480/1035 (46.4) | 855/2170 (39.4) | 348/1035 (33.6) | 585/2170 (27.0) |
| 8 | 498/1053 (47.3) | 902/2200 (41.0) | 467/1053 (44.3) | 791/2200 (36.0) | 331/1053 (31.4) | 550/2200 (25.0) |
| 9 | 485/1058 (45.8) | 855/2224 (38.4) | 436/1058 (41.2) | 743/2224 (33.4) | 281/1058 (26.6) | 499/2224 (22.4) |
| 10 | 432/1027 (42.1) | 762/2169 (35.1) | 387/1027 (37.7) | 667/2169 (30.8) | 242/1027 (23.6) | 445/2169 (20.5) |
| 11 | 423/1035 (40.9) | 721/2205 (32.7) | 361/1035 (34.9) | 616/2205 (27.9) | 217/1035 (21.0) | 409/2205 (18.5) |
| 12 | 392/1008 (38.9) | 649/2156 (30.1) | 310/1008 (30.8) | 588/2156 (27.3) | 211/1008 (20.9) | 378/2156 (17.5) |
| 13 | 371/1025 (36.2) | 602/2158 (27.9) | 307/1025 (30.0) | 553/2158 (25.6) | 192/1025 (18.7) | 333/2158 (15.4) |
| 14 | 332/979 (33.9) | 570/2086 (27.3) | 276/979 (28.2) | 481/2086 (23.1) | 188/979 (19.2) | 317/2086 (15.2) |
| 15 | 282/872 (32.3) | 496/1835 (27.0) | 242/872 (27.8) | 452/1835 (24.6) | 172/872 (19.7) | 278/1835 (15.1) |
| 16 | 311/988 (31.5) | 529/2093 (25.3) | 252/988 (25.5) | 446/2093 (21.3) | 158/988 (16.0) | 295/2093 (14.1) |
| 17 | 280/966 (29.0) | 501/2001 (25.0) | 233/966 (24.1) | 441/2001 (22.0) | 173/966 (17.9) | 282/2001 (14.1) |
| 18 | 281/992 (28.3) | 496/2120 (23.4) | 226/992 (22.8) | 436/2120 (20.6) | 153/992 (15.4) | 269/2120 (12.7) |
| 19 | 252/965 (26.1) | 457/2052 (22.3) | 220/965 (22.8) | 399/2052 (19.4) | 134/965 (13.9) | 242/2052 (11.8) |
| 20 | 239/990 (24.1) | 457/2052 (22.3) | 228/990 (23.0) | 397/2052 (19.3) | 146/990 (14.7) | 235/2052 (11.5) |
| 21 | 248/937 (26.5) | 410/1969 (20.8) | 212/937 (22.6) | 348/1969 (17.7) | 131/937 (14.0) | 205/1969 (10.4) |
| 22 | 218/973 (22.4) | 410/2073 (19.8) | 194/973 (19.9) | 348/2073 (16.8) | 119/973 (12.2) | 209/2073 (10.1) |
| 23 | 216/938 (23.0) | 375/1940 (19.3) | 191/938 (20.4) | 322/1940 (16.6) | 107/938 (11.4) | 197/1940 (10.2) |
| 24 | 188/878 (21.4) | 331/1857 (17.8) | 179/878 (20.4) | 295/1857 (15.9) | 103/878 (11.7) | 191/1857 (10.3) |
| 25 | 188/933 (20.2) | 326/1941 (16.8) | 177/933 (19.0) | 292/1941 (15.0) | 107/933 (11.5) | 192/1941 (9.9) |
| 26 | 162/882 (18.4) | 301/1901 (15.8) | 154/882 (17.5) | 295/1901 (15.5) | 93/882 (10.5) | 169/1901 (8.9) |
| 27 | 154/895 (17.2) | 284/1851 (15.3) | 156/895 (17.4) | 278/1851 (15.0) | 91/895 (10.2) | 168/1851 (9.1) |
| 28 | 146/846 (17.3) | 258/1756 (14.7) | 134/846 (15.8) | 259/1756 (14.7) | 89/846 (10.5) | 146/1756 (8.3) |
| 29 | 123/700 (17.6) | 206/1465 (14.1) | 108/700 (15.4) | 215/1465 (14.7) | 58/700 (8.3) | 110/1465 (7.5) |

| **Day** | **Body aches or joint pain** | | **Loss of taste/smell** | | **Chills** | |
| --- | --- | --- | --- | --- | --- | --- |
|  | **Placebo** | **CAS + IMD** | **Placebo** | **CAS + IMD** | **Placebo** | **CAS + IMD** |
| 1 | 689/1258 (54.8) | 1354/2558 (52.9) | 589/1258 (46.8) | 1123/2558 (43.9) | 471/1258 (37.4) | 898/2558 (35.1) |
| 2 | 506/1161 (43.6) | 949/2353 (40.3) | 580/1161 (50.0) | 1129/2353 (48.0) | 321/1161 (27.6) | 617/2353 (26.2) |
| 3 | 443/1151 (38.5) | 703/2363 (29.8) | 586/1151 (50.9) | 1127/2363 (47.7) | 242/1151 (21.0) | 249/2363 (10.5) |
| 4 | 382/1123 (34.0) | 537/2320 (23.1) | 563/1123 (50.1) | 1079/2320 (46.5) | 184/1123 (16.4) | 175/2320 (7.5) |
| 5 | 328/1119 (29.3) | 474/2316 (20.5) | 560/1119 (50.0) | 1006/2316 (43.4) | 158/1119 (14.1) | 132/2316 (5.7) |
| 6 | 284/1094 (26.0) | 392/2252 (17.4) | 521/1094 (47.6) | 906/2252 (40.2) | 120/1094 (11.0) | 89/2252 (4.0) |
| 7 | 237/1035 (22.9) | 348/2170 (16.0) | 452/1035 (43.7) | 811/2170 (37.4) | 103/1035 (10.0) | 73/2170 (3.4) |
| 8 | 201/1053 (19.1) | 297/2200 (13.5) | 430/1053 (40.8) | 734/2200 (33.4) | 84/1053 (8.0) | 61/2200 (2.8) |
| 9 | 183/1058 (17.3) | 287/2224 (12.9) | 386/1058 (36.5) | 688/2224 (30.9) | 66/1058 (6.2) | 50/2224 (2.2) |
| 10 | 149/1027 (14.5) | 254/2169 (11.7) | 336/1027 (32.7) | 618/2169 (28.5) | 47/1027 (4.6) | 40/2169 (1.8) |
| 11 | 133/1035 (12.9) | 246/2205 (11.2) | 329/1035 (31.8) | 553/2205 (25.1) | 41/1035 (4.0) | 39/2205 (1.8) |
| 12 | 121/1008 (12.0) | 224/2156 (10.4) | 295/1008 (29.3) | 514/2156 (23.8) | 27/1008 (2.7) | 40/2156 (1.9) |
| 13 | 103/1025 (10.0) | 221/2158 (10.2) | 284/1025 (27.7) | 475/2158 (22.0) | 34/1025 (3.3) | 41/2158 (1.9) |
| 14 | 112/979 (11.4) | 216/2086 (10.4) | 247/979 (25.2) | 444/2086 (21.3) | 24/979 (2.5) | 45/2086 (2.2) |
| 15 | 91/872 (10.4) | 184/1835 (10.0) | 195/872 (22.4) | 356/1835 (19.4) | 19/872 (2.2) | 36/1835 (2.0) |
| 16 | 85/988 (8.6) | 198/2093 (9.5) | 227/988 (23.0) | 384/2093 (18.3) | 18/988 (1.8) | 35/2093 (1.7) |
| 17 | 77/966 (8.0) | 183/2001 (9.1) | 204/966 (21.1) | 341/2001 (17.0) | 18/966 (1.9) | 36/2001 (1.8) |
| 18 | 87/992 (8.8) | 175/2120 (8.3) | 191/992 (19.3) | 333/2120 (15.7) | 14/992 (1.4) | 30/2120 (1.4) |
| 19 | 73/965 (7.6) | 166/2052 (8.1) | 186/965 (19.3) | 309/2052 (15.1) | 10/965 (1.0) | 25/2052 (1.2) |
| 20 | 84/990 (8.5) | 167/2052 (8.1) | 178/990 (18.0) | 293/2052 (14.3) | 15/990 (1.5) | 19/2052 (0.9) |
| 21 | 78/937 (8.3) | 161/1969 (8.2) | 159/937 (17.0) | 298/1969 (15.1) | 16/937 (1.7) | 22/1969 (1.1) |
| 22 | 68/973 (7.0) | 157/2073 (7.6) | 157/973 (16.1) | 293/2073 (14.1) | 15/973 (1.5) | 28/2073 (1.4) |
| 23 | 79/938 (8.4) | 148/1940 (7.6) | 145/938 (15.5) | 254/1940 (13.1) | 11/938 (1.2) | 18/1940 (0.9) |
| 24 | 75/878 (8.5) | 141/1857 (7.6) | 149/878 (17.0) | 250/1857 (13.5) | 10/878 (1.1) | 18/1857 (1.0) |
| 25 | 75/933 (8.0) | 136/1941 (7.0) | 144/933 (15.4) | 243/1941 (12.5) | 15/933 (1.6) | 16/1941 (0.8) |
| 26 | 70/882 (7.9) | 139/1901 (7.3) | 131/882 (14.9) | 229/1901 (12.0) | 16/882 (1.8) | 21/1901 (1.1) |
| 27 | 66/895 (7.4) | 135/1851 (7.3) | 124/895 (13.9) | 222/1851 (12.0) | 9/895 (1.0) | 17/1851 (0.9) |
| 28 | 67/846 (7.9) | 111/1756 (6.3) | 118/846 (13.9) | 202/1756 (11.5) | 10/846 (1.2) | 14/1756 (0.8) |
| 29 | 47/700 (6.7) | 92/1465 (6.3) | 87/700 (12.4) | 159/1465 (10.9) | 5/700 (0.7) | 13/1465 (0.9) |

| **Day** | **Runny nose** | | **Sore throat** | | **Loss of appetite** | |
| --- | --- | --- | --- | --- | --- | --- |
|  | **Placebo** | **CAS + IMD** | **Placebo** | **CAS + IMD** | **Placebo** | **CAS + IMD** |
| 1 | 446/1258 (35.5) | 987/2558 (38.6) | 431/1258 (34.3) | 866/2558 (33.9) | 419/1258 (33.3) | 851/2558 (33.3) |
| 2 | 310/1161 (26.7) | 734/2353 (31.2) | 276/1161 (23.8) | 584/2353 (24.8) | 359/1161 (30.9) | 660/2353 (28.0) |
| 3 | 274/1151 (23.8) | 610/2363 (25.8) | 235/1151 (20.4) | 471/2363 (19.9) | 310/1151 (26.9) | 555/2363 (23.5) |
| 4 | 248/1123 (22.1) | 496/2320 (21.4) | 196/1123 (17.5) | 391/2320 (16.9) | 302/1123 (26.9) | 456/2320 (19.7) |
| 5 | 215/1119 (19.2) | 397/2316 (17.1) | 177/1119 (15.8) | 300/2316 (13.0) | 256/1119 (22.9) | 386/2316 (16.7) |
| 6 | 209/1094 (19.1) | 352/2252 (15.6) | 152/1094 (13.9) | 238/2252 (10.6) | 223/1094 (20.4) | 331/2252 (14.7) |
| 7 | 160/1035 (15.5) | 318/2170 (14.7) | 134/1035 (12.9) | 187/2170 (8.6) | 210/1035 (20.3) | 272/2170 (12.5) |
| 8 | 151/1053 (14.3) | 290/2200 (13.2) | 114/1053 (10.8) | 174/2200 (7.9) | 184/1053 (17.5) | 227/2200 (10.3) |
| 9 | 139/1058 (13.1) | 287/2224 (12.9) | 98/1058 (9.3) | 163/2224 (7.3) | 163/1058 (15.4) | 181/2224 (8.1) |
| 10 | 127/1027 (12.4) | 272/2169 (12.5) | 83/1027 (8.1) | 145/2169 (6.7) | 130/1027 (12.7) | 157/2169 (7.2) |
| 11 | 113/1035 (10.9) | 262/2205 (11.9) | 85/1035 (8.2) | 128/2205 (5.8) | 115/1035 (11.1) | 147/2205 (6.7) |
| 12 | 100/1008 (9.9) | 230/2156 (10.7) | 81/1008 (8.0) | 130/2156 (6.0) | 106/1008 (10.5) | 120/2156 (5.6) |
| 13 | 103/1025 (10.0) | 216/2158 (10.0) | 74/1025 (7.2) | 125/2158 (5.8) | 88/1025 (8.6) | 111/2158 (5.1) |
| 14 | 101/979 (10.3) | 200/2086 (9.6) | 69/979 (7.0) | 124/2086 (5.9) | 60/979 (6.1) | 91/2086 (4.4) |
| 15 | 74/872 (8.5) | 166/1835 (9.0) | 68/872 (7.8) | 88/1835 (4.8) | 58/872 (6.7) | 70/1835 (3.8) |
| 16 | 84/988 (8.5) | 171/2093 (8.2) | 72/988 (7.3) | 100/2093 (4.8) | 56/988 (5.7) | 83/2093 (4.0) |
| 17 | 71/966 (7.3) | 160/2001 (8.0) | 63/966 (6.5) | 95/2001 (4.7) | 48/966 (5.0) | 69/2001 (3.4) |
| 18 | 75/992 (7.6) | 146/2120 (6.9) | 58/992 (5.8) | 92/2120 (4.3) | 50/992 (5.0) | 72/2120 (3.4) |
| 19 | 66/965 (6.8) | 141/2052 (6.9) | 59/965 (6.1) | 83/2052 (4.0) | 44/965 (4.6) | 68/2052 (3.3) |
| 20 | 64/990 (6.5) | 151/2052 (7.4) | 52/990 (5.3) | 97/2052 (4.7) | 42/990 (4.2) | 54/2052 (2.6) |
| 21 | 63/937 (6.7) | 144/1969 (7.3) | 49/937 (5.2) | 85/1969 (4.3) | 38/937 (4.1) | 49/1969 (2.5) |
| 22 | 62/973 (6.4) | 131/2073 (6.3) | 43/973 (4.4) | 80/2073 (3.9) | 38/973 (3.9) | 50/2073 (2.4) |
| 23 | 58/938 (6.2) | 132/1940 (6.8) | 47/938 (5.0) | 81/1940 (4.2) | 31/938 (3.3) | 55/1940 (2.8) |
| 24 | 49/878 (5.6) | 114/1857 (6.1) | 41/878 (4.7) | 61/1857 (3.3) | 26/878 (3.0) | 40/1857 (2.2) |
| 25 | 48/933 (5.1) | 112/1941 (5.8) | 42/933 (4.5) | 71/1941 (3.7) | 25/933 (2.7) | 40/1941 (2.1) |
| 26 | 44/882 (5.0) | 105/1901 (5.5) | 32/882 (3.6) | 61/1901 (3.2) | 22/882 (2.5) | 44/1901 (2.3) |
| 27 | 37/895 (4.1) | 107/1851 (5.8) | 33/895 (3.7) | 58/1851 (3.1) | 18/895 (2.0) | 38/1851 (2.1) |
| 28 | 36/846 (4.3) | 95/1756 (5.4) | 33/846 (3.9) | 62/1,756 (3.5) | 20/846 (2.4) | 27/1756 (1.5) |
| 29 | 31/700 (4.4) | 82/1465 (5.6) | 33/700 (4.7) | 45/1465 (3.1) | 18/700 (2.6) | 21/1465 (1.4) |

| **Day** | **Feeling feverish** | | **Sneezing** | | **Shortness of breath** | |
| --- | --- | --- | --- | --- | --- | --- |
|  | **Placebo** | **CAS + IMD** | **Placebo** | **CAS + IMD** | **Placebo** | **CAS + IMD** |
| 1 | 380/1258 (30.2) | 775/2558 (30.3) | 355/1,258 (28.2) | 720/2558 (28.1) | 306/1258 (24.3) | 601/2558 (23.5) |
| 2 | 252/1161 (21.7) | 591/2353 (25.1) | 266/1,161 (22.9) | 575/2353 (24.4) | 250/1161 (21.5) | 474/2353 (20.1) |
| 3 | 239/1151 (20.8) | 220/2363 (9.3) | 240/1,151 (20.9) | 463/2363 (19.6) | 253/1151 (22.0) | 469/2363 (19.8) |
| 4 | 174/1123 (15.5) | 140/2320 (6.0) | 194/1,123 (17.3) | 354/2320 (15.3) | 228/1123 (20.3) | 425/2320 (18.3) |
| 5 | 160/1119 (14.3) | 92/2316 (4.0) | 173/1,119 (15.5) | 284/2316 (12.3) | 233/1119 (20.8) | 376/2316 (16.2) |
| 6 | 128/1094 (11.7) | 84/2252 (3.7) | 141/1,094 (12.9) | 236/2252 (10.5) | 214/1094 (19.6) | 344/2252 (15.3) |
| 7 | 100/1035 (9.7) | 66/2170 (3.0) | 116/1,035 (11.2) | 223/2170 (10.3) | 187/1035 (18.1) | 304/2170 (14.0) |
| 8 | 84/1053 (8.0) | 57/2200 (2.6) | 106/1,053 (10.1) | 193/2200 (8.8) | 189/1053 (17.9) | 283/2200 (12.9) |
| 9 | 69/1058 (6.5) | 41/2224 (1.8) | 103/1,058 (9.7) | 191/2224 (8.6) | 162/1058 (15.3) | 255/2224 (11.5) |
| 10 | 54/1027 (5.3) | 32/2169 (1.5) | 90/1,027 (8.8) | 181/2169 (8.3) | 151/1027 (14.7) | 213/2169 (9.8) |
| 11 | 40/1035 (3.9) | 39/2205 (1.8) | 83/1,035 (8.0) | 169/2205 (7.7) | 141/1035 (13.6) | 206/2205 (9.3) |
| 12 | 25/1008 (2.5) | 30/2156 (1.4) | 73/1,008 (7.2) | 131/2156 (6.1) | 127/1008 (12.6) | 200/2156 (9.3) |
| 13 | 20/1025 (2.0) | 27/2158 (1.3) | 59/1,025 (5.8) | 141/2158 (6.5) | 118/1025 (11.5) | 185/2158 (8.6) |
| 14 | 13/979 (1.3) | 31/2086 (1.5) | 55/979 (5.6) | 133/2086 (6.4) | 104/979 (10.6) | 170/2086 (8.1) |
| 15 | 15/872 (1.7) | 25/1835 (1.4) | 39/872 (4.5) | 109/1835 (5.9) | 96/872 (11.0) | 142/1835 (7.7) |
| 16 | 11/988 (1.1) | 23/2093 (1.1) | 47/988 (4.8) | 117/2093 (5.6) | 96/988 (9.7) | 151/2093 (7.2) |
| 17 | 9/966 (0.9) | 21/2001 (1.0) | 42/966 (4.3) | 115/2001 (5.7) | 86/966 (8.9) | 154/2001 (7.7) |
| 18 | 9/992 (0.9) | 19/2120 (0.9) | 41/992 (4.1) | 113/2120 (5.3) | 91/992 (9.2) | 143/2120 (6.7) |
| 19 | 6/965 (0.6) | 18/2052 (0.9) | 30/965 (3.1) | 91/2052 (4.4) | 97/965 (10.1) | 140/2052 (6.8) |
| 20 | 8/990 (0.8) | 16/2052 (0.8) | 38/990 (3.8) | 94/2052 (4.6) | 97/990 (9.8) | 142/2052 (6.9) |
| 21 | 11/937 (1.2) | 15/1969 (0.8) | 31/937 (3.3) | 96/1969 (4.9) | 89/937 (9.5) | 138/1969 (7.0) |
| 22 | 10/973 (1.0) | 16/2073 (0.8) | 30/973 (3.1) | 102/2073 (4.9) | 92/973 (9.5) | 129/2073 (6.2) |
| 23 | 7/938 (0.7) | 9/1940 (0.5) | 30/938 (3.2) | 89/1940 (4.6) | 83/938 (8.8) | 128/1940 (6.6) |
| 24 | 7/878 (0.8) | 12/1857 (0.6) | 31/878 (3.5) | 87/1857 (4.7) | 76/878 (8.7) | 117/1857 (6.3) |
| 25 | 8/933 (0.9) | 12/1941 (0.6) | 36/933 (3.9) | 81/1941 (4.2) | 75/933 (8.0) | 115/1941 (5.9) |
| 26 | 7/882 (0.8) | 13/1901 (0.7) | 28/882 (3.2) | 70/1901 (3.7) | 71/882 (8.0) | 111/1901 (5.8) |
| 27 | 5/895 (0.6) | 8/1851 (0.4) | 29/895 (3.2) | 66/1851 (3.6) | 70/895 (7.8) | 120/1851 (6.5) |
| 28 | 11/846 (1.3) | 10/1756 (0.6) | 25/846 (3.0) | 65/1756 (3.7) | 65/846 (7.7) | 102/1756 (5.8) |
| 29 | 6/700 (0.9) | 6/1465 (0.4) | 19/700 (2.7) | 51/1465 (3.5) | 50/700 (7.1) | 81/1465 (5.5) |

| **Day** | **Diarrhea** | | **Sputum/phlegm** | | **Nausea** | |
| --- | --- | --- | --- | --- | --- | --- |
|  | **Placebo** | **CAS + IMD** | **Placebo** | **CAS + IMD** | **Placebo** | **CAS + IMD** |
| 1 | 282/1258 (22.4) | 538/2558 (21.0) | 278/1258 (22.1) | 580/2558 (22.7) | 266/1258 (21.1) | 568/2558 (22.2) |
| 2 | 279/1161 (24.0) | 541/2353 (23.0) | 255/1161 (22.0) | 532/2353 (22.6) | 246/1161 (21.2) | 451/2353 (19.2) |
| 3 | 250/1151 (21.7) | 445/2363 (18.8) | 263/1151 (22.8) | 522/2363 (22.1) | 211/1151 (18.3) | 359/2363 (15.2) |
| 4 | 201/1123 (17.9) | 315/2320 (13.6) | 257/1123 (22.9) | 492/2320 (21.2) | 194/1123 (17.3) | 295/2320 (12.7) |
| 5 | 195/1119 (17.4) | 290/2316 (12.5) | 240/1119 (21.4) | 475/2316 (20.5) | 161/1119 (14.4) | 265/2316 (11.4) |
| 6 | 174/1094 (15.9) | 226/2252 (10.0) | 244/1094 (22.3) | 452/2252 (20.1) | 155/1094 (14.2) | 206/2252 (9.1) |
| 7 | 140/1035 (13.5) | 191/2170 (8.8) | 209/1035 (20.2) | 414/2170 (19.1) | 127/1035 (12.3) | 174/2170 (8.0) |
| 8 | 121/1053 (11.5) | 172/2200 (7.8) | 201/1053 (19.1) | 365/2200 (16.6) | 106/1053 (10.1) | 144/2200 (6.5) |
| 9 | 90/1058 (8.5) | 171/2224 (7.7) | 203/1058 (19.2) | 332/2224 (14.9) | 86/1058 (8.1) | 107/2224 (4.8) |
| 10 | 92/1027 (9.0) | 123/2169 (5.7) | 184/1027 (17.9) | 325/2169 (15.0) | 60/1027 (5.8) | 94/2169 (4.3) |
| 11 | 80/1035 (7.7) | 119/2205 (5.4) | 180/1035 (17.4) | 287/2205 (13.0) | 57/1035 (5.5) | 87/2205 (3.9) |
| 12 | 64/1008 (6.3) | 92/2156 (4.3) | 161/1008 (16.0) | 267/2156 (12.4) | 45/1008 (4.5) | 73/2156 (3.4) |
| 13 | 63/1025 (6.1) | 85/2158 (3.9) | 154/1025 (15.0) | 237/2158 (11.0) | 39/1025 (3.8) | 65/2158 (3.0) |
| 14 | 50/979 (5.1) | 71/2086 (3.4) | 127/979 (13.0) | 222/2086 (10.6) | 38/979 (3.9) | 47/2086 (2.3) |
| 15 | 43/872 (4.9) | 53/1835 (2.9) | 111/872 (12.7) | 178/1835 (9.7) | 19/872 (2.2) | 45/1835 (2.5) |
| 16 | 40/988 (4.0) | 71/2093 (3.4) | 126/988 (12.8) | 214/2093 (10.2) | 30/988 (3.0) | 52/2093 (2.5) |
| 17 | 40/966 (4.1) | 61/2001 (3.0) | 118/966 (12.2) | 195/2001 (9.7) | 24/966 (2.5) | 48/2001 (2.4) |
| 18 | 36/992 (3.6) | 63/2120 (3.0) | 121/992 (12.2) | 206/2120 (9.7) | 20/992 (2.0) | 48/2120 (2.3) |
| 19 | 28/965 (2.9) | 52/2052 (2.5) | 116/965 (12.0) | 191/2052 (9.3) | 15/965 (1.6) | 35/2052 (1.7) |
| 20 | 29/990 (2.9) | 44/2052 (2.1) | 108/990 (10.9) | 189/2052 (9.2) | 14/990 (1.4) | 43/2052 (2.1) |
| 21 | 31/937 (3.3) | 37/1969 (1.9) | 98/937 (10.5) | 170/1969 (8.6) | 16/937 (1.7) | 38/1969 (1.9) |
| 22 | 28/973 (2.9) | 35/2073 (1.7) | 90/973 (9.2) | 168/2073 (8.1) | 22/973 (2.3) | 35/2073 (1.7) |
| 23 | 18/938 (1.9) | 37/1940 (1.9) | 77/938 (8.2) | 158/1940 (8.1) | 12/938 (1.3) | 31/1940 (1.6) |
| 24 | 20/878 (2.3) | 24/1857 (1.3) | 80/878 (9.1) | 140/1857 (7.5) | 18/878 (2.1) | 25/1857 (1.3) |
| 25 | 19/933 (2.0) | 32/1941 (1.6) | 78/933 (8.4) | 138/1941 (7.1) | 14/933 (1.5) | 33/1941 (1.7) |
| 26 | 20/882 (2.3) | 23/1901 (1.2) | 73/882 (8.3) | 148/1901 (7.8) | 11/882 (1.2) | 27/1901 (1.4) |
| 27 | 17/895 (1.9) | 33/1851 (1.8) | 62/895 (6.9) | 130/1851 (7.0) | 10/895 (1.1) | 26/1851 (1.4) |
| 28 | 16/846 (1.9) | 22/1756 (1.3) | 64/846 (7.6) | 115/1756 (6.5) | 8/846 (0.9) | 19/1756 (1.1) |
| 29 | 15/700 (2.1) | 15/1465 (1.0) | 47/700 (6.7) | 99/1465 (6.8) | 5/700 (0.7) | 15/1465 (1.0) |

| **Day** | **Red or watery eyes** | | **Dizziness** | | **Pressure/tightness in chest** | |
| --- | --- | --- | --- | --- | --- | --- |
|  | **Placebo** | **CAS + IMD** | **Placebo** | **CAS + IMD** | **Placebo** | **CAS + IMD** |
| 1 | 228/1258 (18.1) | 510/2558 (19.9) | 219/1258 (17.4) | 450/2558 (17.6) | 199/1258 (15.8) | 436/2558 (17.0) |
| 2 | 148/1161 (12.7) | 370/2353 (15.7) | 158/1161 (13.6) | 320/2353 (13.6) | 163/1161 (14.0) | 298/2353 (12.7) |
| 3 | 146/1151 (12.7) | 262/2363 (11.1) | 151/1151 (13.1) | 304/2363 (12.9) | 151/1151 (13.1) | 256/2363 (10.8) |
| 4 | 105/1123 (9.3) | 222/2320 (9.6) | 144/1123 (12.8) | 244/2320 (10.5) | 145/1123 (12.9) | 210/2320 (9.1) |
| 5 | 100/1119 (8.9) | 194/2316 (8.4) | 110/1119 (9.8) | 222/2316 (9.6) | 134/1119 (12.0) | 184/2316 (7.9) |
| 6 | 77/1094 (7.0) | 163/2252 (7.2) | 106/1094 (9.7) | 174/2252 (7.7) | 123/1094 (11.2) | 163/2252 (7.2) |
| 7 | 57/1035 (5.5) | 138/2170 (6.4) | 97/1035 (9.4) | 148/2170 (6.8) | 104/1035 (10.0) | 154/2170 (7.1) |
| 8 | 58/1053 (5.5) | 134/2200 (6.1) | 90/1053 (8.5) | 136/2200 (6.2) | 97/1053 (9.2) | 129/2200 (5.9) |
| 9 | 57/1058 (5.4) | 134/2224 (6.0) | 70/1058 (6.6) | 116/2224 (5.2) | 68/1058 (6.4) | 127/2224 (5.7) |
| 10 | 43/1027 (4.2) | 116/2169 (5.3) | 56/1027 (5.5) | 87/2169 (4.0) | 72/1027 (7.0) | 102/2169 (4.7) |
| 11 | 41/1035 (4.0) | 105/2205 (4.8) | 60/1035 (5.8) | 95/2205 (4.3) | 67/1035 (6.5) | 93/2205 (4.2) |
| 12 | 34/1008 (3.4) | 99/2156 (4.6) | 55/1008 (5.5) | 87/2156 (4.0) | 66/1008 (6.5) | 103/2156 (4.8) |
| 13 | 33/1025 (3.2) | 90/2158 (4.2) | 49/1025 (4.8) | 93/2158 (4.3) | 62/1025 (6.0) | 85/2158 (3.9) |
| 14 | 32/979 (3.3) | 76/2086 (3.6) | 50/979 (5.1) | 76/2086 (3.6) | 53/979 (5.4) | 83/2086 (4.0) |
| 15 | 27/872 (3.1) | 63/1835 (3.4) | 39/872 (4.5) | 63/1835 (3.4) | 41/872 (4.7) | 70/1835 (3.8) |
| 16 | 29/988 (2.9) | 72/2093 (3.4) | 45/988 (4.6) | 74/2093 (3.5) | 44/988 (4.5) | 79/2093 (3.8) |
| 17 | 33/966 (3.4) | 74/2001 (3.7) | 40/966 (4.1) | 64/2001 (3.2) | 36/966 (3.7) | 64/2001 (3.2) |
| 18 | 27/992 (2.7) | 72/2120 (3.4) | 38/992 (3.8) | 65/2120 (3.1) | 52/992 (5.2) | 70/2120 (3.3) |
| 19 | 21/965 (2.2) | 67/2052 (3.3) | 31/965 (3.2) | 70/2052 (3.4) | 48/965 (5.0) | 59/2052 (2.9) |
| 20 | 19/990 (1.9) | 64/2052 (3.1) | 31/990 (3.1) | 60/2052 (2.9) | 45/990 (4.5) | 65/2052 (3.2) |
| 21 | 22/937 (2.3) | 64/1969 (3.3) | 27/937 (2.9) | 45/1969 (2.3) | 43/937 (4.6) | 66/1969 (3.4) |
| 22 | 22/973 (2.3) | 60/2073 (2.9) | 24/973 (2.5) | 40/2073 (1.9) | 52/973 (5.3) | 63/2073 (3.0) |
| 23 | 25/938 (2.7) | 66/1940 (3.4) | 28/938 (3.0) | 40/1940 (2.1) | 40/938 (4.3) | 59/1940 (3.0) |
| 24 | 22/878 (2.5) | 55/1857 (3.0) | 23/878 (2.6) | 37/1857 (2.0) | 38/878 (4.3) | 49/1857 (2.6) |
| 25 | 23/933 (2.5) | 56/1941 (2.9) | 22/933 (2.4) | 32/1941 (1.6) | 38/933 (4.1) | 44/1941 (2.3) |
| 26 | 17/882 (1.9) | 54/1901 (2.8) | 25/882 (2.8) | 33/1901 (1.7) | 33/882 (3.7) | 50/1901 (2.6) |
| 27 | 14/895 (1.6) | 57/1851 (3.1) | 20/895 (2.2) | 36/1851 (1.9) | 41/895 (4.6) | 58/1851 (3.1) |
| 28 | 16/846 (1.9) | 43/1756 (2.4) | 18/846 (2.1) | 28/1756 (1.6) | 31/846 (3.7) | 36/1756 (2.1) |
| 29 | 11/700 (1.6) | 35/1465 (2.4) | 18/700 (2.6) | 27/1465 (1.8) | 17/700 (2.4) | 32/1465 (2.2) |

| **Day** | **Stomach ache** | | **Chest pain** | | **Confusion** | |
| --- | --- | --- | --- | --- | --- | --- |
|  | **Placebo** | **CAS + IMD** | **Placebo** | **CAS + IMD** | **Placebo** | **CAS + IMD** |
| 1 | 162/1258 (12.9) | 312/2558 (12.2) | 92/1258 (7.3) | 200/2558 (7.8) | 89/1258 (7.1) | 176/2558 (6.9) |
| 2 | 114/1161 (9.8) | 246/2353 (10.5) | 76/1161 (6.5) | 122/2353 (5.2) | 55/1161 (4.7) | 113/2353 (4.8) |
| 3 | 110/1151 (9.6) | 200/2363 (8.5) | 63/1151 (5.5) | 102/2363 (4.3) | 36/1151 (3.1) | 89/2363 (3.8) |
| 4 | 97/1123 (8.6) | 169/2320 (7.3) | 59/1123 (5.3) | 99/2320 (4.3) | 36/1123 (3.2) | 81/2320 (3.5) |
| 5 | 90/1119 (8.0) | 145/2316 (6.3) | 52/1119 (4.6) | 67/2316 (2.9) | 37/1119 (3.3) | 72/2316 (3.1) |
| 6 | 83/1094 (7.6) | 115/2252 (5.1) | 36/1094 (3.3) | 65/2252 (2.9) | 27/1094 (2.5) | 56/2252 (2.5) |
| 7 | 60/1035 (5.8) | 85/2170 (3.9) | 37/1035 (3.6) | 61/2170 (2.8) | 32/1035 (3.1) | 52/2170 (2.4) |
| 8 | 54/1,053 (5.1) | 87/2200 (4.0) | 44/1053 (4.2) | 38/2200 (1.7) | 32/1053 (3.0) | 52/2200 (2.4) |
| 9 | 46/1058 (4.3) | 75/2224 (3.4) | 36/1058 (3.4) | 41/2224 (1.8) | 23/1058 (2.2) | 46/2224 (2.1) |
| 10 | 39/1027 (3.8) | 65/2169 (3.0) | 37/1027 (3.6) | 35/2169 (1.6) | 24/1027 (2.3) | 36/2169 (1.7) |
| 11 | 44/1035 (4.3) | 54/2205 (2.4) | 28/1035 (2.7) | 33/2205 (1.5) | 27/1035 (2.6) | 39/2205 (1.8) |
| 12 | 37/1008 (3.7) | 49/2156 (2.3) | 23/1008 (2.3) | 41/2156 (1.9) | 17/1008 (1.7) | 40/2156 (1.9) |
| 13 | 31/1025 (3.0) | 52/2158 (2.4) | 25/1025 (2.4) | 38/2158 (1.8) | 17/1025 (1.7) | 34/2158 (1.6) |
| 14 | 26/979 (2.7) | 46/2086 (2.2) | 18/979 (1.8) | 41/2086 (2.0) | 19/979 (1.9) | 34/2086 (1.6) |
| 15 | 26/872 (3.0) | 43/1835 (2.3) | 22/872 (2.5) | 25/1835 (1.4) | 16/872 (1.8) | 29/1835 (1.6) |
| 16 | 31/988 (3.1) | 40/2093 (1.9) | 21/988 (2.1) | 36/2093 (1.7) | 20/988 (2.0) | 33/2093 (1.6) |
| 17 | 25/966 (2.6) | 35/2001 (1.7) | 19/966 (2.0) | 40/2001 (2.0) | 19/966 (2.0) | 31/2001 (1.5) |
| 18 | 25/992 (2.5) | 33/2120 (1.6) | 22/992 (2.2) | 36/2120 (1.7) | 18/992 (1.8) | 30/2120 (1.4) |
| 19 | 26/965 (2.7) | 32/2052 (1.6) | 18/965 (1.9) | 29/2052 (1.4) | 14/965 (1.5) | 21/2052 (1.0) |
| 20 | 23/990 (2.3) | 31/2052 (1.5) | 20/990 (2.0) | 30/2052 (1.5) | 13/990 (1.3) | 27/2052 (1.3) |
| 21 | 23/937 (2.5) | 34/1969 (1.7) | 20/937 (2.1) | 34/,969 (1.7) | 17/937 (1.8) | 19/1969 (1.0) |
| 22 | 19/973 (2.0) | 34/2073 (1.6) | 14/973 (1.4) | 32/2073 (1.5) | 12/973 (1.2) | 25/2073 (1.2) |
| 23 | 18/938 (1.9) | 33/1940 (1.7) | 20/938 (2.1) | 26/1940 (1.3) | 17/938 (1.8) | 20/1940 (1.0) |
| 24 | 19/878 (2.2) | 22/1857 (1.2) | 19/878 (2.2) | 25/1857 (1.3) | 15/878 (1.7) | 20/1857 (1.1) |
| 25 | 16/933 (1.7) | 36/1941 (1.9) | 15/933 (1.6) | 20/1941 (1.0) | 14/933 (1.5) | 21/1941 (1.1) |
| 26 | 20/882 (2.3) | 32/1901 (1.7) | 14/882 (1.6) | 21/1901 (1.1) | 13/882 (1.5) | 24/1901 (1.3) |
| 27 | 18/895 (2.0) | 25/1851 (1.4) | 15/895 (1.7) | 27/1851 (1.5) | 10/895 (1.1) | 21/1851 (1.1) |
| 28 | 7/846 (0.8) | 19/1756 (1.1) | 13/846 (1.5) | 26/1756 (1.5) | 7/846 (0.8) | 19/1756 (1.1) |
| 29 | 11/700 (1.6) | 16/1465 (1.1) | 13/700 (1.9) | 18/1465 (1.2) | 11/700 (1.6) | 16/1465 (1.1) |

| **Day** | **Vomiting** | | **Rash** | |
| --- | --- | --- | --- | --- |
|  | **Placebo** | **CAS + IMD** | **Placebo** | **CAS + IMD** |
| 1 | 50/1258 (4.0) | 89/2558 (3.5) | 20/1258 (1.6) | 54/2558 (2.1) |
| 2 | 32/1161 (2.8) | 68/2353 (2.9) | 16/1161 (1.4) | 51/2353 (2.2) |
| 3 | 32/1151 (2.8) | 32/2363 (1.4) | 15/1151 (1.3) | 44/2363 (1.9) |
| 4 | 34/1123 (3.0) | 30/2320 (1.3) | 15/1123 (1.3) | 32/2320 (1.4) |
| 5 | 21/1119 (1.9) | 24/2316 (1.0) | 19/1119 (1.7) | 35/2316 (1.5) |
| 6 | 19/1094 (1.7) | 13/2252 (0.6) | 17/1094 (1.6) | 36/2252 (1.6) |
| 7 | 21/1035 (2.0) | 11/2170 (0.5) | 18/1035 (1.7) | 27/2170 (1.2) |
| 8 | 19/1053 (1.8) | 12/2200 (0.5) | 13/1053 (1.2) | 25/2200 (1.1) |
| 9 | 13/1058 (1.2) | 11/2224 (0.5) | 15/1058 (1.4) | 29/2224 (1.3) |
| 10 | 8/1027 (0.8) | 8/2169 (0.4) | 12/1027 (1.2) | 24/2169 (1.1) |
| 11 | 6/1035 (0.6) | 9/2205 (0.4) | 13/1035 (1.3) | 18/2205 (0.8) |
| 12 | 8/1008 (0.8) | 12/2156 (0.6) | 9/1008 (0.9) | 22/2156 (1.0) |
| 13 | 6/1025 (0.6) | 6/2158 (0.3) | 10/1025 (1.0) | 20/2158 (0.9) |
| 14 | 5/979 (0.5) | 5/2086 (0.2) | 9/979 (0.9) | 17/2086 (0.8) |
| 15 | 5/872 (0.6) | 4/1835 (0.2) | 10/872 (1.1) | 14/1835 (0.8) |
| 16 | 4/988 (0.4) | 3/2093 (0.1) | 6/988 (0.6) | 17/2093 (0.8) |
| 17 | 4/966 (0.4) | 6/2001 (0.3) | 11/966 (1.1) | 15/2001 (0.7) |
| 18 | 5/992 (0.5) | 6/2120 (0.3) | 11/992 (1.1) | 15/2120 (0.7) |
| 19 | 3/965 (0.3) | 5/2052 (0.2) | 8/965 (0.8) | 14/2052 (0.7) |
| 20 | 5/990 (0.5) | 6/2052 (0.3) | 9/990 (0.9) | 22/2052 (1.1) |
| 21 | 2/937 (0.2) | 3/1969 (0.2) | 7/937 (0.7) | 19/1969 (1.0) |
| 22 | 4/973 (0.4) | 3/2073 (0.1) | 9/973 (0.9) | 16/2073 (0.8) |
| 23 | 6/938 (0.6) | 3/1,940 (0.2) | 10/938 (1.1) | 14/1940 (0.7) |
| 24 | 2/878 (0.2) | 2/1857 (0.1) | 12/878 (1.4) | 13/1857 (0.7) |
| 25 | 3/933 (0.3) | 4/1941 (0.2) | 9/933 (1.0) | 16/1941 (0.8) |
| 26 | 4/882 (0.5) | 2/1901 (0.1) | 5/882 (0.6) | 19/1901 (1.0) |
| 27 | 2/895 (0.2) | 2/1851 (0.1) | 6/895 (0.7) | 12/1851 (0.6) |
| 28 | 3/846 (0.4) | 4/1756 (0.2) | 10/846 (1.2) | 15/1756 (0.9) |
| 29 | 3/700 (0.4) | 0/1465 (0.0) | 4/700 (0.6) | 10/1465 (0.7) |

The proportion of patients with symptoms is defined as the number of patients with any level of severity (i.e., mild/moderate/severe) of the indicated symptom divided by total number of patients with data at each day (day 1–29).

CAS + IMD, casirivimab and imdevimab.

Supplemental Figure 1. Treatment effect trajectories by study cohort


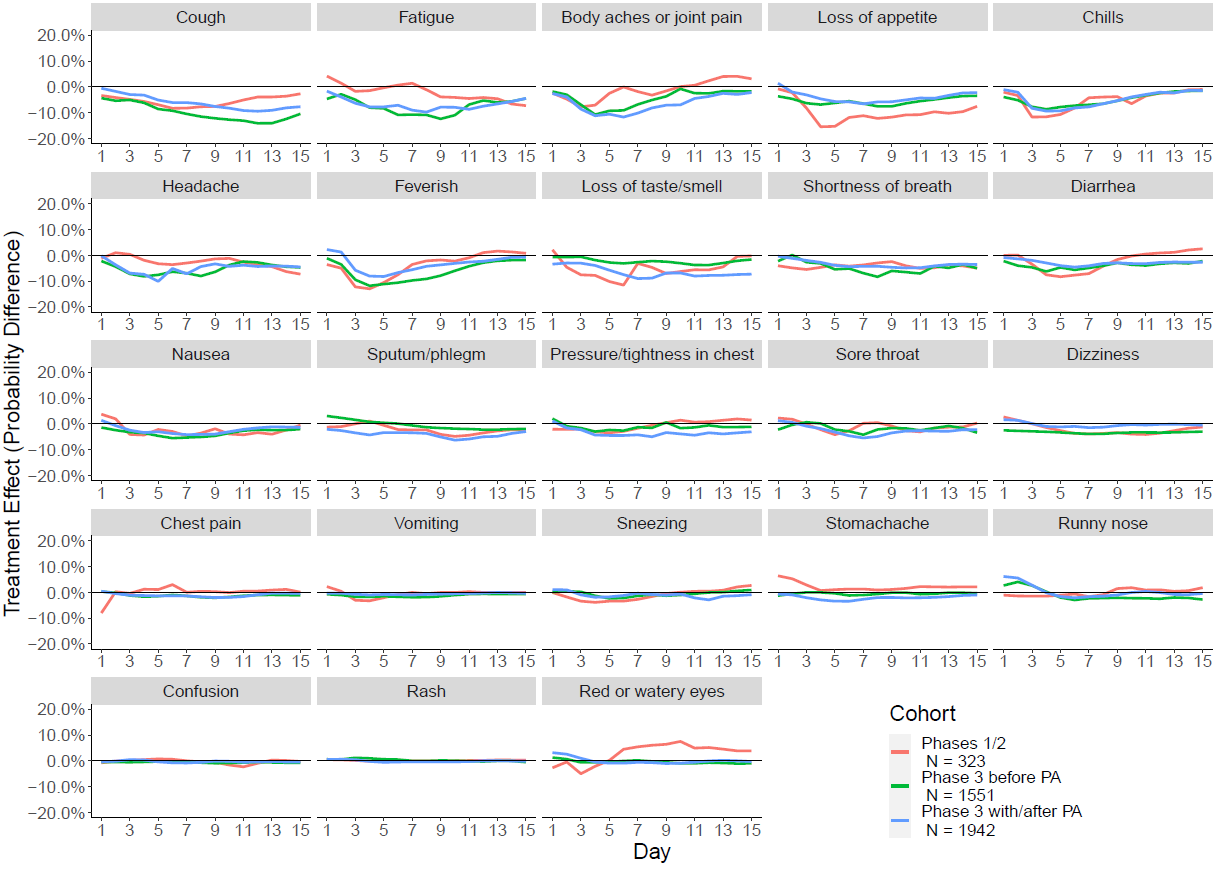


Treatment effect trajectories for each symptom of the Symptoms Evolution of COVID-19 instrument were obtained using a 2-step approach (see Statistical Methodology for more details) and analyzed by study cohort (Phase I/II, Phase III before PA, and Phase III with/after PA).

PA, protocol amendment.

Supplemental Figure 2. Treatment effect trajectories in patients without risk factors for hospitalization


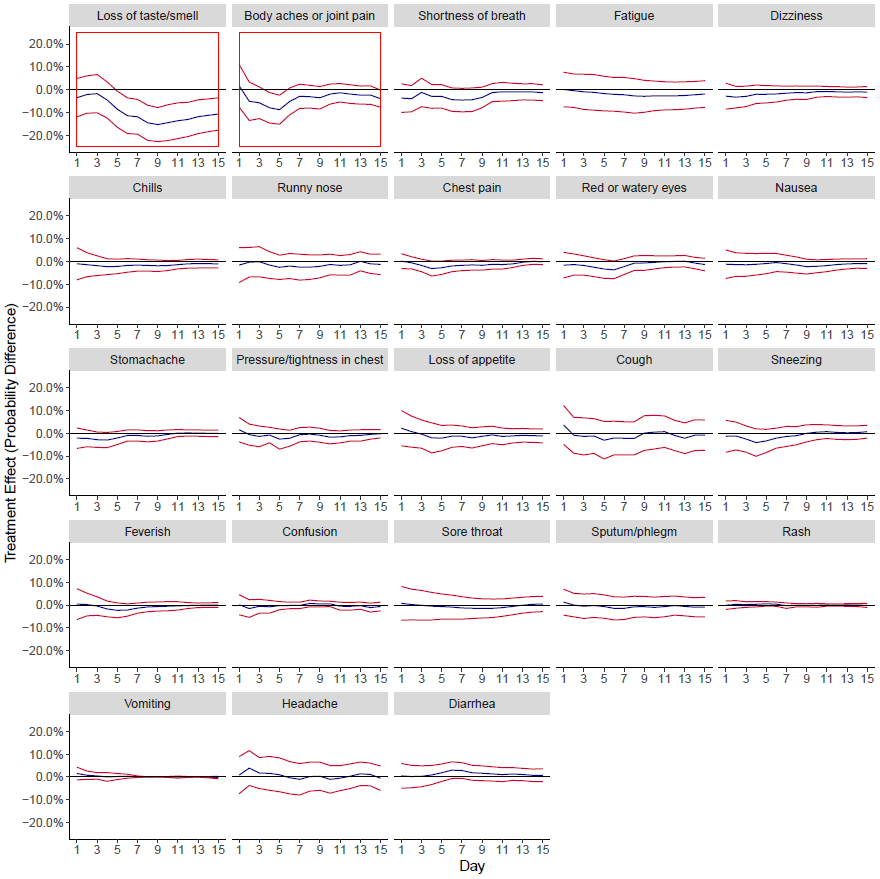


Treatment effect trajectories for each symptom of the Symptoms Evolution of COVID-19 instrument were obtained using a 2-step approach (see Statistical Methodology for more details). Curve estimates are indicated by the blue lines in the center and 95% confidence bands by the red lines. Symptoms with significant treatment effects are highlighted in red boxes (upper bounds of the confidence bands below 0 for at least 2 consecutive days). The symptoms are ranked by area under the curve for the treatment effect trajectories relative to horizontal line y = 0.0%.

Supplemental Figure 3. Hierarchical clustering of symptoms experienced by patients in the adaptive Phase I/II/III CAS + IMD clinical trial. (A) Hierarchical clustering was performed to group the 17 symptoms with significant treatment effects over time. An optimal number of clusters was determined using gap statistics. (B) A cluster dendrogram shows each pair of symptoms that was analyzed to generate composite symptom variables

**A**
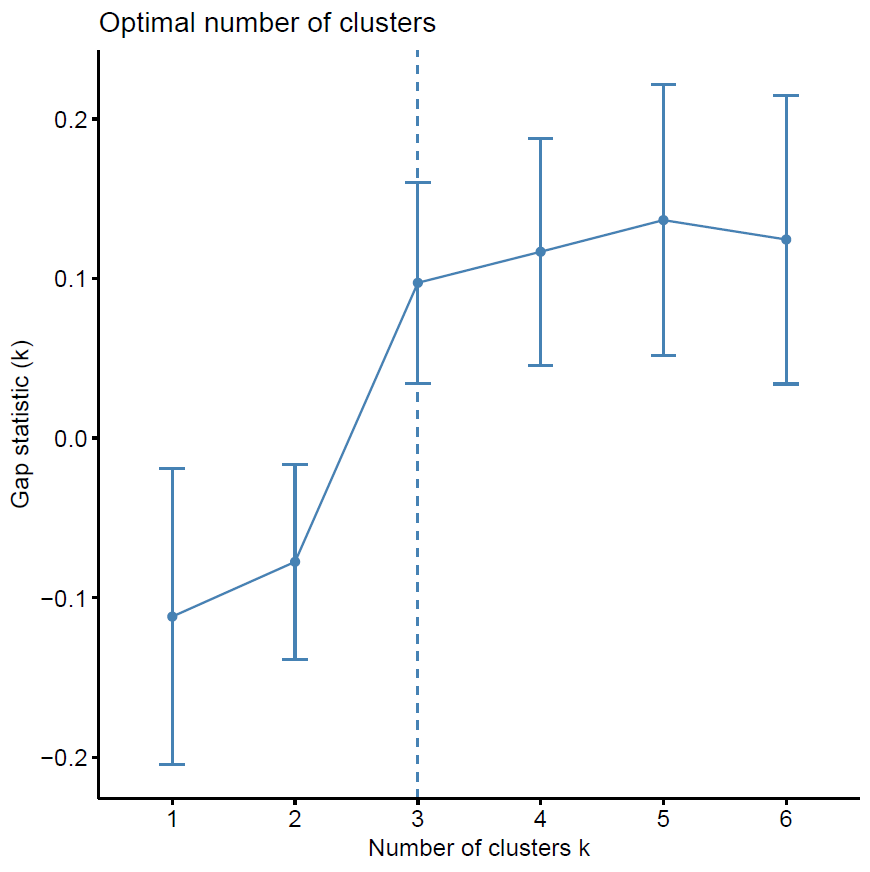


**B**

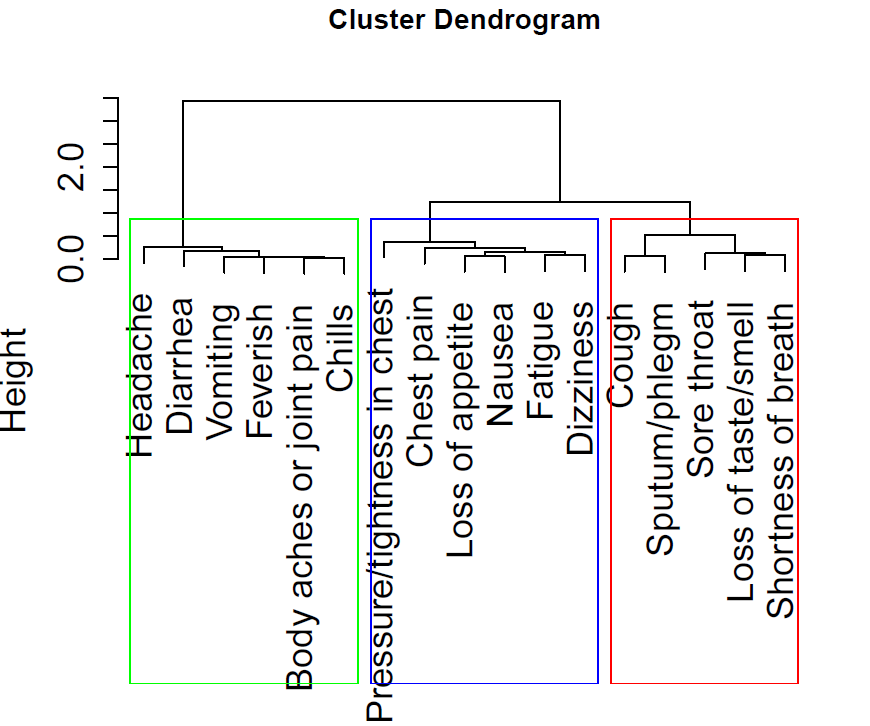


(For each pair of symptoms, similarity between treatment trajectories was quantified by 1 – Pearson correlation.

Green = cluster 1; blue = cluster 2; red = cluster 3.

CAS + IMD, casirivimab and imdevimab.

Supplemental Figure 4. Evolution of symptoms in the placebo arm by baseline antibody profile. (A) IgA, (B) IgG, (C) NAb, and (D) seroconversion

**A**

**
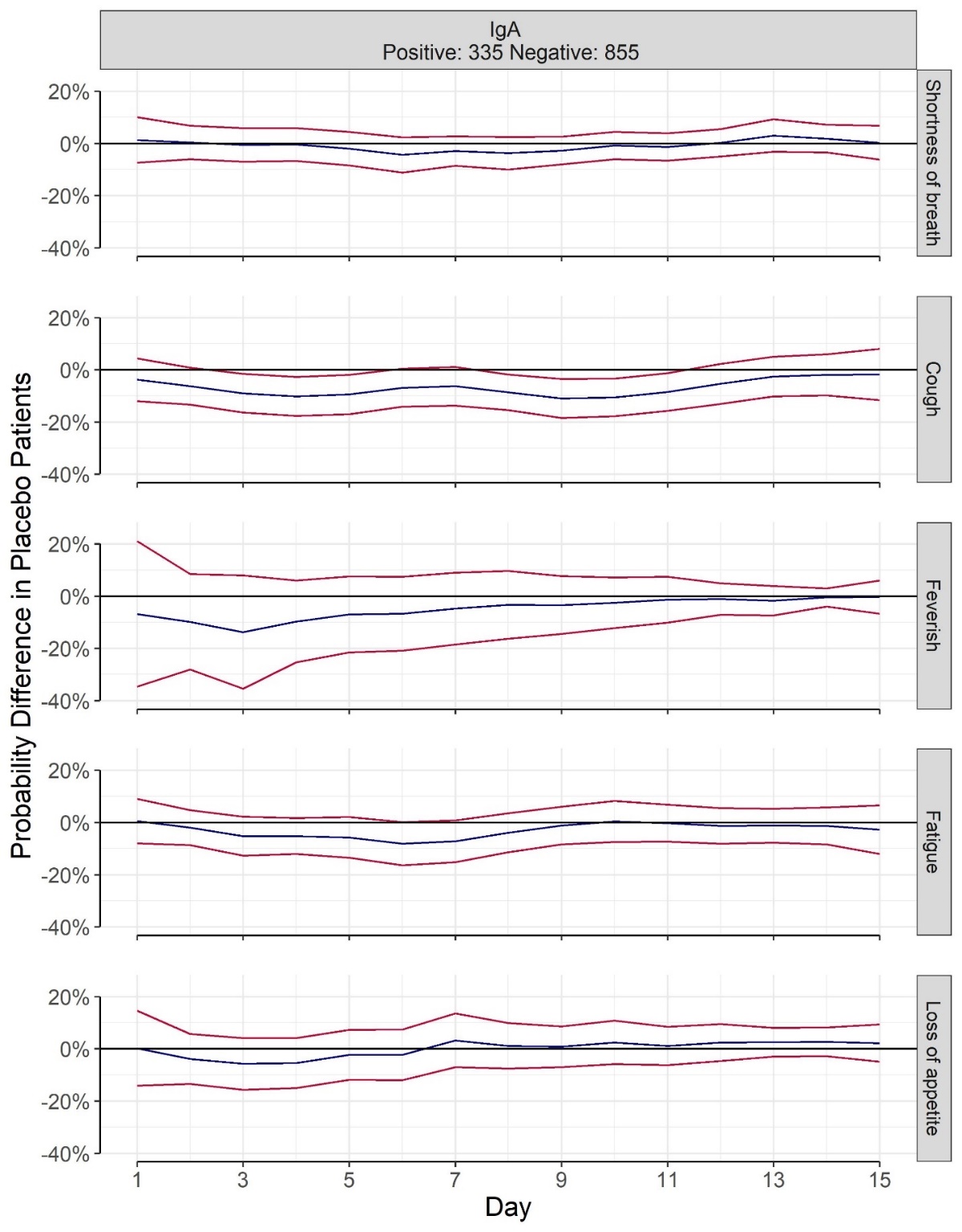
**

**B**

**
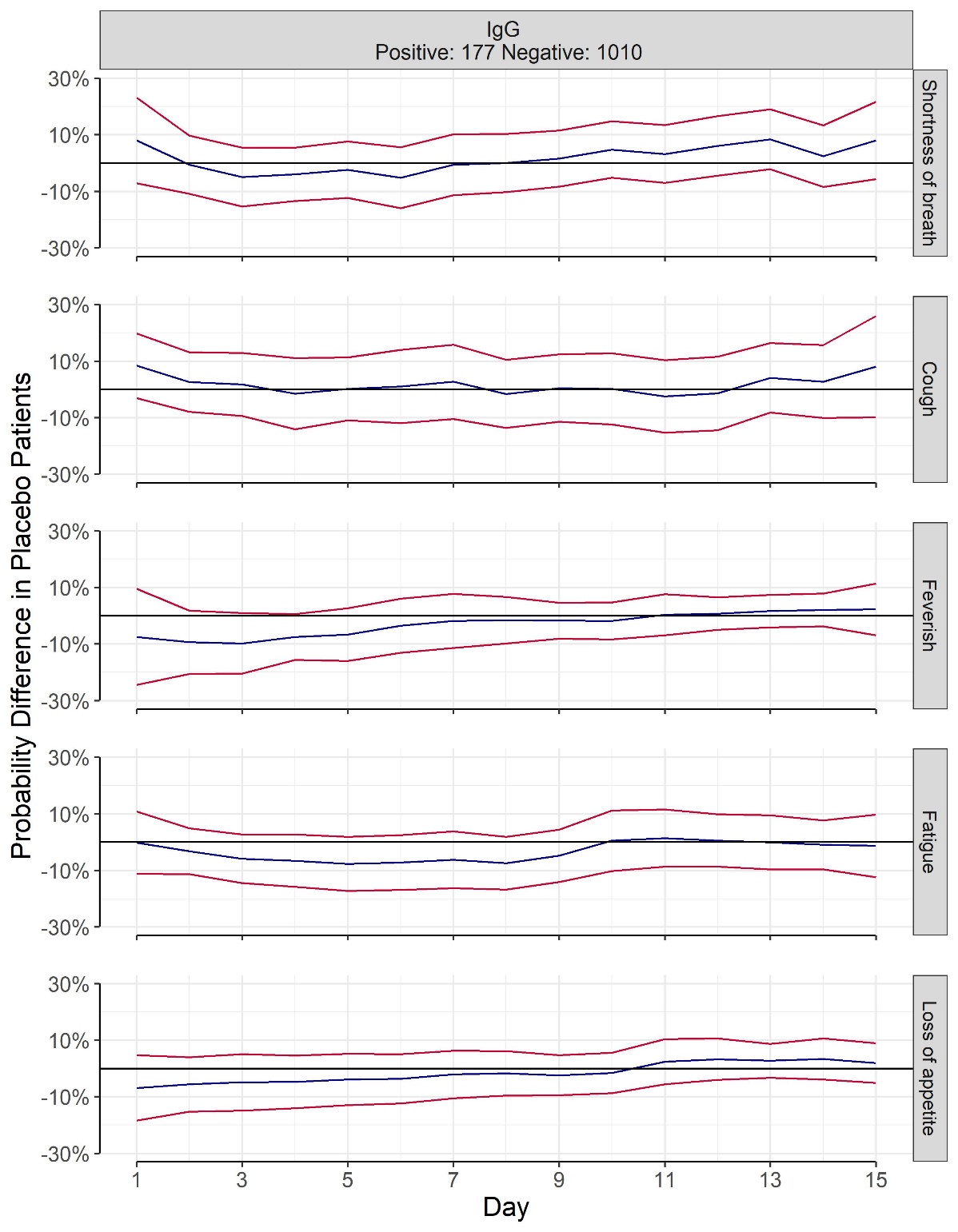
**

**C**


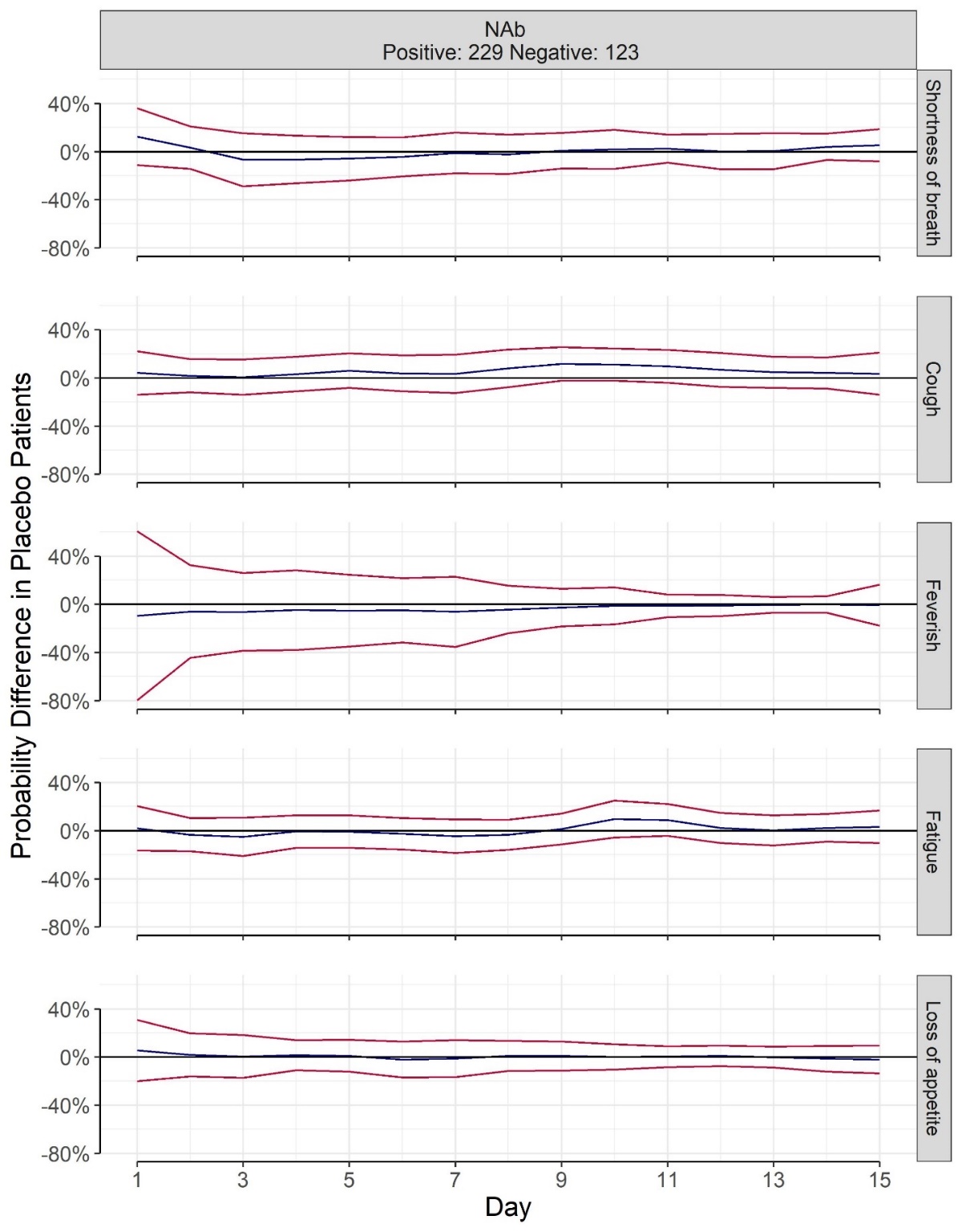


**D**


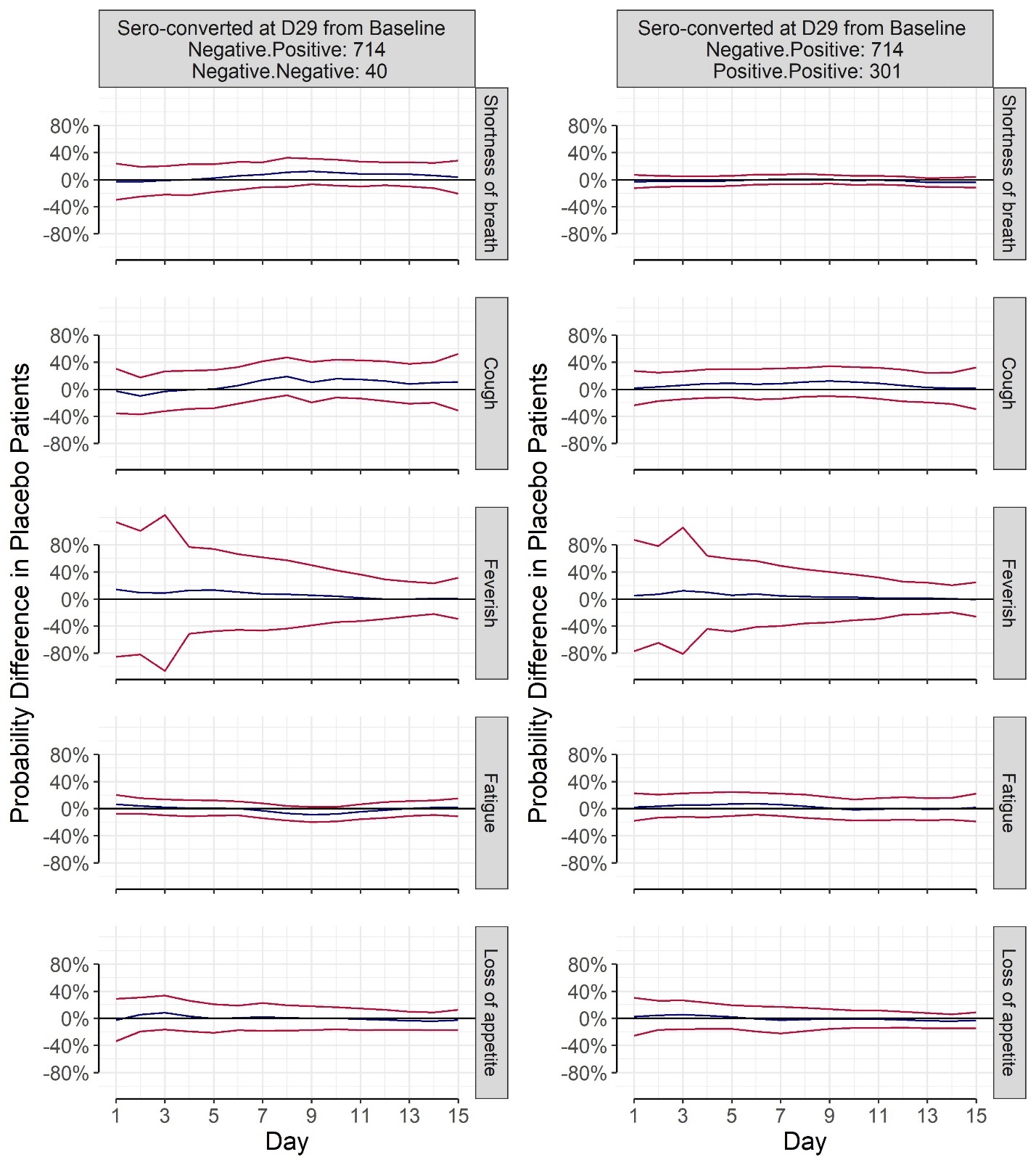


Negative.Positive: Seronegative at baseline and anti-N IgG-positive at day 29; Negative.Negative: Seronegative at baseline and anti-N IgG-negative at day 29; Positive.Positive: Seropositive at baseline and anti-N IgG-positive at day 29.

Trajectories for each symptom of the Symptoms Evolution of COVID-19 instrument were obtained using a 2-step approach (see Statistical Methodology for more details). The curve estimate is indicated by the blue line in the center, and 95% confidence bands are indicated by the red lines.

D, day; NAb, neutralizing antibody.
